# Microglia Express Insulin-Like Growth Factor-1 in the Hippocampus of Aged APP_swe_/PS1_ΔE9_ Transgenic Mice

**DOI:** 10.3389/fncel.2019.00308

**Published:** 2019-07-30

**Authors:** Christa Løth Myhre, Camilla Thygesen, Birgitte Villadsen, Jeanette Vollerup, Laura Ilkjær, Katrine Tækker Krohn, Manuela Grebing, Shuainan Zhao, Asif Manzoor Khan, Lasse Dissing-Olesen, Morten Skovgaard Jensen, Alicia A. Babcock, Bente Finsen

**Affiliations:** ^1^Department of Neurobiology, Institute of Molecular Medicine, University of Southern Denmark, Odense, Denmark; ^2^Brain Research – Inter-Disciplinary Guided Excellence, Department of Clinical Research, University of Southern Denmark, Odense, Denmark; ^3^Department of Biochemistry and Molecular Biology, University of Southern Denmark, Odense, Denmark; ^4^Department of Biomedicine, Aarhus University, Aarhus, Denmark

**Keywords:** neuroinflammation, tumor necrosis factor, insulin-like growth factor, cerebral amyloidosis, aging, neurogenesis

## Abstract

Insulin-like growth factor-1 (IGF-1) is a pleiotropic molecule with neurotrophic and immunomodulatory functions. Knowing the capacity of chronically activated microglia to produce IGF-1 may therefore show essential to promote beneficial microglial functions in Alzheimer’s disease (AD). Here, we investigated the expression of IGF-1 mRNA and IGF-1 along with the expression of tumor necrosis factor (TNF) mRNA, and the amyloid-β (Aβ) plaque load in the hippocampus of 3- to 24-month-old APP_swe_/PS1_ΔE9_ transgenic (Tg) and wild-type (WT) mice. As IGF-1, in particular, is implicated in neurogenesis we also monitored the proliferation of cells in the subgranular zone (sgz) of the dentate gyrus. We found that the Aβ plaque load reached its maximum in aged 21- and 24-month-old APP_swe_/PS1_ΔE9_ Tg mice, and that microglial reactivity and hippocampal IGF-1 and TNF mRNA levels were significantly elevated in aged APP_swe_/PS1_ΔE9_ Tg mice. The sgz cell proliferation decreased with age, regardless of genotype and increased IGF-1/TNF mRNA levels. Interestingly, IGF-1 mRNA was expressed in subsets of sgz cells, likely neuroblasts, and neurons in both genotypes, regardless of age, as well as in glial-like cells. By double *in situ* hybridization these were shown to be IGF1 mRNA^+^ CD11b mRNA^+^ cells, i.e., IGF-1 mRNA-expressing microglia. Quantification showed a 2-fold increase in the number of microglia and IGF-1 mRNA-expressing microglia in the molecular layer of the dentate gyrus in aged APP_swe_/PS1_ΔE9_ Tg mice. Double-immunofluorescence showed that IGF-1 was expressed in a subset of Aβ plaque-associated CD11b^+^ microglia and in several subsets of neurons. Exposure of primary murine microglia and BV2 cells to Aβ_42_ did not affect IGF-1 mRNA expression. IGF-1 mRNA levels remained constant in WT mice with aging, unlike TNF mRNA levels which increased with aging. In conclusion, our results suggest that the increased IGF-1 mRNA levels can be ascribed to a larger number of IGF-1 mRNA-expressing microglia in the aged APP_swe_/PS1_ΔE9_ Tg mice. The finding that subsets of microglia retain the capacity to express IGF-1 mRNA and IGF-1 in the aged APP_swe_/PS1_ΔE9_ Tg mice is encouraging, considering the beneficial therapeutic potential of modulating microglial production of IGF-1 in AD.

## Introduction

Alzheimer’s disease (AD) is an age-associated progressive neurodegenerative disease and the most common cause of dementia. Histopathological changes include accumulation of beta-amyloid (Aβ) in plaques and a chronic microglial reaction, that takes its onset years before a diagnosis can be made (Cagnin et al., 2001; Heneka et al., 2015, Heppner et al., 2015). Knowing microglial capacity to produce neurotrophic and immunomodulatory factors, such as the insulin-like growth factor-1 (IGF-1) (Fernandez and Torres-Alemán, 2012), as well as factors with potential deleterious functions, such as the pleiotrophic cytokine tumor necrosis factor (TNF) (Arnett et al., 2001; Lambertsen et al., 2009), is therefore essential to promote beneficial and ameliorate detrimental microglial functions. While it is well-known that microglia are a major source of TNF in transgenic mouse models of AD (Hickman et al., 2008; Minogue et al., 2014; Babcock et al., 2015), there is less information about microglia as a significant local source of IGF-1. To our knowledge, the first evidence of microglia being a source of IGF-1 in the adult CNS was the finding of a transient deafferentation-induced up-regulation of IGF-1 mRNA in the perforant pathway innervated parts of the dentate gyrus in young, adult rats which functionally was suggested to be involved in deafferentation-induced axonal sprouting (Guthrie et al., 1995). This up-regulation of IGF-1 mRNA was reported to be attenuated in aged rats (Woods et al., 1998).

IGF-1 is a member of the insulin gene family. It is a 70 amino acid long growth factor hormone with potent anabolic effects during development (Arroba et al., 2018). Deficiency in IGF-1 leads to microcephalus and mental retardation in the human (Woods et al., 1996). IGF-1 is transiently expressed in high levels in central projection neurons during development (Bondy, 1991; Bondy et al., 1992), consistent with an important role in brain development and neuroplasticity (Dyer et al., 2016). Additionally, an IGF-1 mRNA-expressing microglial subset was recently reported to support myelinogenesis during development (Wlodarczyk et al., 2017). IGF-1 binds with high affinity to the IGF-1 receptor (IGF-1R) as well as the insulin receptor (lower affinity), which are both tyrosine kinase receptors, sharing signaling molecules and trophic activities (Fernandez and Torres-Alemán, 2012). While the expression of IGF-1 declines with development, the IGF-1R, remains expressed at high level in the adult brain (Bondy et al., 1992).

Aβ plaque load has been shown to follow a sigmoidal path in the neocortex of both AD patients (Serrano-Pozo et al., 2011; Jack et al., 2013) and in the APP_swe_/PS1_ΔE9_ transgenic (Tg) mouse (Jankowsky et al., 2004; Babcock et al., 2015), eventually stabilizing and reaching a plateau. Whether the same phenomenon occurs in the hippocampus is unknown. Aβ plaques appear in the hippocampus during early stages of AD (Thal et al., 2002), especially within the target zone of the perforant pathway carrying information from the entorhinal cortex into the hippocampus (Hyman et al., 1986; Pooler et al., 2013). Unlike in normal aging, AD patients lose hippocampal CA1 neurons (West et al., 1994), and hippocampal atrophy is associated with the development of AD (Bobinski et al., 2000; Nelson et al., 2012; Huijbers et al., 2015). The hippocampus is also a site of neurogenesis, which involves proliferation of cells in the subgranular zone (sgz) of the dentate gyrus (Eriksson et al., 1998; Kempermann et al., 2004). Studies report both an increase (Jin et al., 2004) and a decrease (Ziabreva et al., 2006; Crews et al., 2010) in sgz cell proliferation in AD brains, indicating that neurogenesis may be deregulated. Contradictory results on sgz cell proliferation have also been reported in different transgenic mouse models of AD (Chuang, 2010). The APP_swe_/PS1_ΔE9_ Tg mouse, which develops Aβ plaques in the hippocampus from 3 months of age (Hamilton and Holscher, 2012), has been reported to have more pronounced loss of sgz cell proliferation with age than age-matched wild-type (WT) mice by some groups (Demars et al., 2010; Valero et al., 2011; Hamilton and Holscher, 2012). Results by us showed a reduced number of neuroblasts in the sgz of male 18-month-old APP_swe_/PS1_ΔE9_ Tg mice, while sgz cell proliferation was unaffected by genotype (Olesen et al., 2017).

Neurogenesis has been reported to be regulated by both systemic (Foster et al., 2011) and locally-produced IGF-1 (Anderson et al., 2002; Stranahan et al., 2009), and it is reduced during normal aging (Kuhn et al., 1996; Knoth et al., 2010; Spalding et al., 2013). In the case of TNF, small amounts of TNF have been shown to increase sgz cell proliferation, while higher TNF levels can induce apoptosis (Iosif et al., 2006). Decreased serum levels of IGF-1 (Ramsey et al., 2004; Sonntag et al., 2005; Duron et al., 2014), and increased serum levels of TNF in AD patients (Perry et al., 2001; Holmes et al., 2009; Swardfager et al., 2010), might potentially impair hippocampal neurogenesis and/or neuroblast survival and differentiation.

The APP_swe_/PS1_ΔE9_ Tg mouse exhibits changes in both the IGF-1 and TNF system (Zhang et al., 2013; Francois et al., 2014; Minogue et al., 2014; Babcock et al., 2015). Using this mouse model, we recently showed that TNF mRNA levels in the neocortex correlate to the age-dependent increase in Aβ plaque-load as well as aging, and additionally, that microglial production of TNF was functionally correlated to microglial uptake of Aβ (Babcock et al., 2015). We here expanded this study to include an investigation of the accumulation of Aβ plaques, microglial reactivity, and expression of IGF-1 mRNA and IGF-1 as well as TNF mRNA progression with age in the hippocampus of APP_swe_/PS1_ΔE9_ Tg mice. We additionally used double *in situ* hybridization (*ISH*) to investigate whether or not IGF-1 mRNA might co-localize to CD11b mRNA^+^ microglia, besides being expressed in neurons. Finally, we investigated how Aβ pathology and age affected proliferation of sgz cells in the neurogenic niche of the hippocampus. Our results show that a subset of microglia in the aging APP_swe_/PS1_ΔE9_ Tg mouse retain the capacity to express IGF-1 mRNA, suggesting that the increased IGF-1 mRNA levels in the aged APP_swe_/PS1_ΔE9_ Tg mice may be ascribed to microglia. This finding attracts attention to the perspectives of modulating microglial synthesis of IGF-1 in AD.

## Materials and Methods

### Mice and Experimental Material

Female APP_swe_/PS1_ΔE9_ Tg mice that expressed humanized APP (Mo/HuAPP695sweK595N/M596L) and mutant human PS1_ΔE9_ in neurons (Jankowsky et al., 2004) and WT littermates were bred and maintained on a hybrid (C57BL/6 × C3H/HeN; B6C3) background. Additionally, three 24-26-month-old APP_swe_/PS1_ΔE9_ Tg and two 26-month-old WT littermate bred on a C57BL/6 background and two postnatal day 5 (P5) C57BL/6 pups were used for *ISH* and IGF-1 protein detection. All mice were housed and bred in the Biomedical Laboratory, University of Southern Denmark. APP_swe_/PS1_ΔE9_ Tg and WT mice on a hybrid background were perfused with phosphate-buffered saline (PBS) and analyzed at 3, 6, 9, 12, 15, 18, 21, and 24 months of age (*n* = 6–10 per group). Sections (20-μm-thick) were cut from fresh frozen left hemispheres, on a cryostat, and used for immunohistochemistry (IHC) and *ISH*. This experimental material was previously used to quantify changes in Aβ plaque load and TNF mRNA^+^ and interleukin-1β mRNA^+^ cells in the neocortex (Babcock et al., 2015). The hippocampus was dissected from the right hemisphere, and stored in Trizol at −80°C for polymerase chain reaction (qPCR) analysis. Additional mice used for *ISH* were euthanized by decapitation and the brains processed in 20-μm-thick cryostat sections. WT and APP_swe_/PS1_ΔE9_ Tg mice used for double immunofluorescence staining were perfused with Sorensen’s buffer, followed by 4% paraformaldehyde (PFA). After 2 h post-fixation in 4% PFA and overnight immersion in 20% sucrose, these brains were frozen in CO_2_ snow and cut as 20-μm-thick horizontal sections on a cryostat. The proliferation of sgz cells was evaluated in groups of 3-, 9-, and 15-month-old Tg and WT mice receiving 90 mg/kg BrdU i.p. at 2, 12, and 22 h prior to PBS-perfusion (*n* = 6–8 per group). Hippocampi were isolated, immersion-fixed in 4% PFA, followed by 1% PFA and 20% sucrose in each solution overnight at 4°C, and then frozen in CO_2_ snow. Hippocampi were cut into 30-μm-thick cryostat sections. Experiments were conducted according to permission from the Danish Ethical Animal Care Committee (Permissions no. 2011/562-67 and 2011/561-1950).

### Primary Microglia and Microglial BV2 Cells

Primary murine microglia were cultured and isolated as described in Thygesen et al. (2018). Primary microglia were harvested from mixed glia cultures and plated in 24-well culture plates at a density of 1.5 × 10^5^ cells/mL. The BV2 murine microglial cell line was kindly provided by Jan Thorleif Pedersen, Lundbeck A/S, Denmark. Cells were grown in Dulbecco’s modified eagle medium, 10% fetal bovine serum (FBS), 1% penicillin/glutamine/streptomycin in 5% CO_2_ at 37°C and plated in 24-well culture plates at a density of 0.75 × 10^5^ cells/mL. After plating, cells were allowed to settle for 24 h after which they were stimulated with 1 μM Aβ_42_ (Anaspec) for 24 h in 5% CO_2_ at 37°C. The Aβ_42_ peptide solution was prepared according to Stine et al. (2003). Briefly, a lyophilized peptide stock of 0.1 mg was dissolved in dimethyl sulfoxide to a final concentration of 5 mM and diluted to 100 μM in Dulbecco’s modified eagle medium, 10% FBS, 1% penicillin/glutamine/streptomycin, and left 24 h at 37°C to allow peptide aggregation prior to cell stimulation.

### Immunohistochemistry (IHC) and Immunofluorescence Staining

#### Antibodies and Isotype Controls

Biotinylated mouse anti-human Aβ_1–16_, (clone 6e10, Covance), rat anti-mouse CD11b (clone 5C6, Serotec), rat anti-BrdU (clone BU1/75 (ICR1), Abcam), and rabbit anti-IGF-1 (ab9572, Abcam) were used as primary antibodies. Biotinylated goat anti-rat IgG (GE Healthcare United Kingdom limited) and biotinylated goat anti-rat IgG (Thermo) were used as secondary antibodies for BrdU and CD11b IHC, respectively, while an alkaline phosphatase (AP)-conjugated goat-anti rabbit antibody was used for IGF-1 (Sigma, A3812) IHC. Horseradish peroxidase-conjugated streptavidin (HRP-SA) (Dako) was applied after biotinylated antibodies were bound. Biotinylated mouse IgG1 (Caltag), rat IgG2b (Biosite), rat IgG2a (BioLegend), and rabbit IgG (DAKO) were used as isotype or IgG controls.

#### BrdU-Pretreatment

Sections from immersion-fixed hippocampi were stained for BrdU. After post-fixing sections for 10 min in 4% PFA, sections were rinsed with 2 × saline sodium citrate (2 × SSC) and 49% formamide in tris-buffered saline (TBS) for 2 h at 60°C. Sections were then rinsed with 2 × SSC at 60°C for 2 h and incubated with 2N HCl for 2 h at 37°C. Finally, sections were rinsed with 0.1 M sodium borate buffer (pH = 8.5) for 10 min, before proceeding with the IHC protocol.

#### Protocol Aβ, CD11b, and BrdU

IHC was performed as previously described (Babcock et al., 2015). All sections were acclimatized for 30 min at room temperature (RT). Fresh frozen sections to be stained for CD11b were fixed in 4% buffered formalin (pH 7.0) for 2 min, then immersed in 50% acetone, 100% acetone, and 50% acetone for 2 min in each solution, and for Aβ, sections were fixed in 4% PFA overnight. All sections for Aβ, CD11b and BrdU IHC were then rinsed with TBS and TBS + 1% Triton X-100. Non-specific binding was blocked by incubation in TBS containing 10% FBS for 30 min. Primary antibodies or isotype controls were prepared in TBS with 10% FBS and applied for 1 h at RT, followed by 4°C overnight. After acclimatization and rinsing in TBS, endogenous peroxidase was blocked in sections prepared for Aβ and CD11b IHC using 10% methanol and 10% H_2_O_2_ in TBS for 10 min. After rinsing in TBS, secondary antibodies for CD11b and BrdU staining were added for 1 h at RT in TBS containing 10% FBS. SA-conjugated horseradish peroxidase diluted in TBS containing 10% FBS (1:200) was added for 1 h at RT, and then sections were rinsed in TBS and developed with 0.05% diaminobenzidine (DAB) in TBS containing 0.01% H_2_O_2_. Sections were further rinsed in TBS, dehydrated in graded ethanol, cleared in xylene, and coverslipped with Depex mounting medium. BrdU-stained sections and a parallel series of sections were stained with Toluidine blue (pH 7.4), and rinsed three times with H_2_O before dehydration and mounting. No staining was observed in sections stained with isotype controls (data not shown).

#### IHC for IGF-1

IHC for IGF-1 was performed as described for TNF in Lambertsen et al. (2001), however, sections were fixed in 4% PFA overnight, or as described above for CD11b, by use of an AP-conjugated secondary antibody.

#### Immunofluorescence

Immunofluorescence staining was performed in tissue sections from PFA-perfused mice, using a combination of antibodies directed against Aβ (clone 6e10) and CD11b (clone 5C6). The staining procedure was largely as described for IHC (section “Protocol Aβ, CD11b, and BrdU”), except that primary antibodies were applied in combination and steps to block endogenous peroxidase activity were omitted. The bound primary antibodies were detected by incubating sections with SA-TRITC (AbDSerotec) and AlexaFluor488-labeled goat-anti rat IgG (Invitrogen) simultaneously. Sections were kept in the dark after application of secondary reagents. Sections incubated with isotype controls (biotinylated mouse IgG1 (Caltag) or rat IgG2b (Biosite) instead of primary antibodies, showed no staining (data not shown). Immunofluorescence for IGF-1 and CD11b (clone 5C6) was performed on fresh frozen tissue sections from 24-month-old APP_swe_/PS1_ΔE9_ (*n* = 2) and 26-month-old WT (*n* = 1) mice. Sections were brought to RT for 30 min, fixed as described for CD11b, and dried 60 min at RT. The staining was performed as described above, however, using AlexaFluor488-labeled goat-anti rabbit IgG (Invitrogen) and AlexaFluor568 goat-anti rat IgG (Invitrogen) simultaneously as secondary reagents. Specificity was controlled by substitution of the primary antibodies with rabbit IgG and isotype and rat IgG2b. Sections were mounted with DAKO fluorescence mounting medium. Immunofluorescence images were captured with an Olympus BX63 upright, automated fluorescence microscope installed with an Olympus DP80 camera, X-cite 120LED system with the following filter cubes (U-FBNA FL Ex.BP470- 495 Em.BA510-550, U-FGNA FL Ex.BP540-550 EM.BA575-625, U-FMCHE FL Ex.BP 565-585 Em.BA600-690, and U-FUNA FL Ex.BP360-370 EmBA420-460), and objectives (UPLSAPO2 10X/0.4, UPLSAPO2 40X/0.95, PLAPON0 60X/1.42, and UPLSAPO 100X/1.4), using the CellSens Software (Thygesen et al., 2018).

### Estimation of % Aβ Plaque Load and Measurement of Hippocampal Volume

#### % Aβ Plaque Load

The % Aβ plaque load was quantified by estimating the area of the hippocampus covered by Aβ plaques using unbiased stereological principles. This was carried out using the same Aβ-stained sections previously used for quantifying neocortical Aβ plaque load (Babcock et al., 2015). All Aβ plaques were counted by the same person (CM) using an Olympus BX 50-microscope (Olympus, Germany) fitted with a U-PMTVC Japan color camera (Olympus, Germany), a Proscan Prior motorized specimen stage, and a Heidenhain MT12 microcator connected to a PC installed with the CAST-2 Software (Visiopharm, Denmark). The hippocampus in the left hemisphere was delineated in Aβ-stained sections using anatomical borders. After delineation, the software determined the area of the hippocampus that was used for the sampling (A_*HC*_). We used a point-counting method with a 286.5 μm × 216 μm frame and 36-crosses, each corresponding to an area of 1,714.5 μm^2^, which produced an acceptable coefficient of error (CE) of <0.10 in a 9-month-old APP_swe_/PS1_ΔE9_ Tg mouse with moderate Aβ plaque formation. Only Aβ plaques marked by a counting cross in the hippocampus were counted. Counting was carried out systematically in 5–8 sections per mouse. The percentage of hippocampus covered by plaques, % Aβ plaque load, was calculated as previously described (Babcock et al., 2015). The CE for individual mice and the average CE for each age group were calculated as outlined in West et al. (1996). The coefficient of variation, CV, is calculated as SD/Mean. CV and average CE are shown in Supplementary Table S1 for each age group.

#### Hippocampal Volume Estimation

We estimated the volume of the left hippocampus based on the hippocampal areas (A_*HC*_) delineated during Aβ quantification (section “% Aβ Plaque Load”). For each mouse, hippocampal volume (V_*HC*_) was determined using volumetric principles, based on typically four systematically sampled sections, and was calculated using the following equation: V*_*HC*_* = A_*HC*_× *d*, where A*_*HC*_* is the area of the hippocampus (see section “% Aβ Plaque Load”) and *d* is the distance between sections (960 μm). The CV (SD/mean) values for individual age groups are included in Supplementary Table S1.

### qPCR

RNA was isolated using the Trizol method and then converted into cDNA (Babcock et al., 2006; Wirenfeldt et al., 2007). The quantitative PCR (qPCR) reaction was carried out in triplicate in 96-well plates using an Applied Biosystems PRISM 7300 Real time PCR machine (Babcock et al., 2006). Primer and probe sequences used were: for HPRT (F′ GTT AAG CAG TAC AGC CCC AAA ATG, R′ AAA TCC AAC AAA GTC TGG CCT GTA and probe: Fam-AGC TTG CTG GTG AAA AGG ACC TCT CGA AGT); GAPDH (F′ TGT CAA GCT CAT TTC CTG GTA TGA, R′ CTT ACT CCT TGG AGG CCA TGT AG and probe: FAM-TCC ACC ACC CTG TTG CTG TAG CCG); TNF (F′ TGG CCT CCC TCT CAT CAG TTC, R′ CCA CTT GGT GGT TTG CTA CGA and probe: 5′-FAM-TGG CCC AGA CCC TCA CAC TCA GAT CAT C) (Fenger et al., 2006; Meldgaard et al., 2006); IGF-1 (F′ CCG AGG GGC TTT TAC TTC AAC AA, R′CGG AAG CAA CAC TCA TCC ACA A); and brain-derived neurotrophic factor (BDNF) (F′ GGC CCA ACG AAG AAA ACC AT, R′ AGC ATC ACC CGG GAA GTG T). Maxima Probe Master (Fermentas) was used as master mix for TaqMan qPCR (TNF, HPRT, GAPDH) and Maxima Sybr Green Master (Fermentas) as master mix for Sybr Green qPCR (IGF-1, BDNF). In the case of the hippocampal tissue, the qPCR results were calibrated relative to spleen cDNA and were normalized using HPRT, which is stably expressed in mice under pathological conditions in brain tissue (Meldgaard et al., 2006). In the case of the *in vitro* experiments, RNA was isolated with the RNeasy mini kit and the qPCR results were normalized using two reference genes (GAPDH and HPRT), as done in Thygesen et al. (2018). The relative qPCR values on the hippocampal data are shown as fold-increases versus the 3-month-old WT group, on primary microglia as fold-increases compared to whole neocortex, and as Aβ-stimulated versus un-stimulated cells.

### ISH

#### AP-Labeled Probes and Controls

ISH was performed on fresh frozen cryostat sections using a *de novo* synthesized AP-labeled oligo DNA probe specific for IGF-1 mRNA (5′/AP/CCC CTC GGT CCA CAC ACG AAC TGA AGA) or a mixture of two AP-labeled oligo DNA probes specific for TNF mRNA (5′/AP/CG TAG TCG GGG CAG CCT TGT CCC TTG AA and 5′/AP/CT TCT CAT CCC TTT GGG GAC CGA TCA CC) (Gregersen et al., 2000; Lambertsen et al., 2009; Babcock et al., 2015). The IGF-1 probe was designed in Oligo v. 6 to target both IGF-1 isoforms and with sequence specificity verified by BLAST. No specific signal was detected for either system when a series of negative controls was performed. This included pre-treatment of sections with RNAse prior to hybridization in order to control for binding to RNA, hybridization with 100-fold excess of unlabelled probe to control for non-specific binding of the AP-linker arm, and buffer controls (Clausen et al., 2013). A probe for GAPDH mRNA was included as a control for the *ISH* procedure and to know the tissue quality.

##### ISH protocol

Fresh frozen sections were dried at 55°C for 10 min, then dehydrated in 96% Ethanol for 3 h at RT and dried 1 h at RT. Probes mixed in hybridization buffer were applied to sections with coverslips and allowed to hybridize overnight in a dark hybridization chamber at 37°C. Sections were rinsed three times 30 min with 1 × SSC (pH = 9.5) in 55°C preheated holders and rinsed twice with Tris–HCL buffer for 10 min at RT. Sections were developed with Nitro blue tetrazolium (NBT) + 5-bromo-4-chloro-3-indolyl-phosphate (BCIP), for 3 days in the dark at RT. The sections were rinsed in 25°C distilled water for 1 h to stop development, and then mounted with Aquatex.

#### Double-ISH

ViewRNA Tissue Assay Kit (Affymetrix) and probes specific for IGF-1 mRNA (VB1-20972 (Probe 1-AP)) and CD11b (Itgam) mRNA (VB6-15396 (Probe 6-AP)) (Affymetrix) were used for co-expression analysis which, with smaller modifications, was performed according to the QuantiGene ViewRNA ISH Tissue 2-Plex Assay Protocol by Affymetrix as described in Grebing et al. (2016). After ISH the sections were counterstained with hematoxylin (Sigma-Aldrich), rinsed in tap water three times, dried for 1 h and coverslipped in Ultramount (Dako).

### Counting of BrdU^+^ Cells

BrdU^+^ cells were systematically counted in every 10th section of the hippocampus (typically 12–18 sections per mouse), using an Olympus BX 41-microscope (Olympus, Denmark) equipped with a 20 × objective. BrdU^+^ cells were only counted if their nucleus was located in the sgz. The total number of sections containing the hippocampus was determined in a parallel series of Toluidine Blue-stained sections. Data are presented as the mean number of BrdU^+^ cells per hippocampal section.

### Counting of IGF-1 mRNA^+^ CD11b mRNA^+^ Cells

All hematoxylin-stained nuclei were counted manually in the region of interest (ROI) which was either the lateral or medial blade of the molecular layer in the dentate gyrus, together with all CD11b mRNA^+^ and/or IGF-1 mRNA^+^ cells. Quantification was performed in 5 hippocampi in three sections from 3 Tg mice and 3 hippocampi in two sections from 2 WT mice by the use of a 40 × high numerical objective (NA 0.95) and an Olympus BX41 microscope. For a cell to be counted as an either IGF-1 or CD11b mRNA^+^ cell, or as an IGF-1 mRNA^+^ CD11b mRNA^+^ cell, the cell defined by its hematoxylin-stained nucleus, should display one or more red and/or blue puncta, each puncta reflecting the presence of an IGF-1 or CD11b mRNA molecule. To calculate the cellular density the area within which the cells were counted was measured using a microscope Olympus DP80 Dual Color Monochrome CCD camera mounted on a motorized BX63 Olympus microscope and the Olympus CellSens software.

### Statistics

Data are presented graphically with indication of the medians and 25 and 75% quartiles. All statistical analyses were performed using Prism (GraphPad Software, version 6). The % Aβ plaque load and volumetric data were analyzed by Kruskal–Wallis test followed by Dunn’s multiple comparison test, and with linear regression and Boltzmann sigmoidal curve fitting. qPCR and BrdU data were analyzed by Kruskal–Wallis test followed by Dunn’s multiple comparison test. Pearson correlation was performed to investigate relationships between Aβ plaque load, age, and cytokine mRNA levels. Mann–Whitney test was used for comparisons of two groups. Statistically significant differences are indicated as *p* < 0.05^*^, *p* < 0.01^∗∗^, *p* < 0.001^∗∗∗^, and *p* < 0.0001^*⁣*⁣**^.

## Results

### Aβ Plaque Load Follows a Sigmoidal Trajectory in the Hippocampus With Age

**FIGURE 1:**
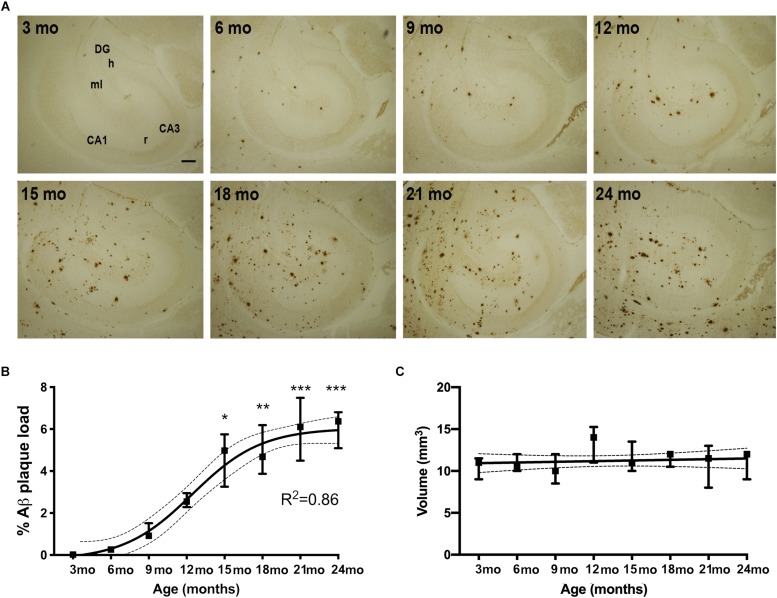
% Aβ plaque load follows a sigmoidal trajectory with age in the hippocampus of APP_swe_/PS1_ΔE9_ Tg mice. **(A)** IHC staining for Aβ shows an age-dependent increase in Aβ plaques in the hippocampus of Tg mice. Initially plaques become abundant in the perforant pathway innervated parts or the hippocampus. CA1, CA3, regio superior and inferior hippocampus, respectively; DG, dentate gyrus; h, hilus; ml, molecular layer; r, stratum radiatum. Scale bar: 100 μm. **(B,C)** Stereological quantification showing that hippocampal % Aβ plaque load increases significantly from 15 months of age and follows a sigmoidal trajectory, based on non-linear regression analyses **(B)**, whereas hippocampal volume is not significantly changed with age in Tg mice **(C)**. 95% confidence intervals are included. Data points show medians and 25 and 75% quartiles for each group [*n* = 6/group except for *n* = 4 for 15-month-old mice in **(B)**, and *n* = 5 or 6/group except for *n* = 4 for 15-month-old mice and *n* = 3 for 24-month-old mice in **(C)**]. Asterisks represent statistically significant increases in % Aβ plaque load versus 3-month-old mice as determined by Dunn’s test. ^*^p < 0.05, ^∗∗^*p* < 0.01, ^∗∗∗^*p* < 0.001.

We first examined the accumulation of Aβ plaques in the hippocampus of APP_swe_/PS1_ΔE9_ Tg mice with age. We visualized Aβ plaques in 3-, 6-, 9-, 12-, 15-, 18-, 21-, and 24-month-old mice using IHC (Figure 1A). Very few Aβ plaques were observed in the hippocampus at 3 months of age. At 6 months of age, Aβ plaques were observed in the molecular layer of the dentate gyrus, with most Aβ plaques located in the terminal area of the perforant pathway, and essentially no Aβ plaques in the dentate hilus or stratum radiatum of the hippocampus proper (Figure 1A). From 9 and 12 months of age, Aβ plaques increased moderately, remaining most frequent in areas innervated by the perforant pathway, with still very few Aβ plaques in the hilus. In the oldest mice, Aβ plaques were found in all hippocampal regions (Figure 1A). To quantify % Aβ plaque load in the entire hippocampus, we used unbiased stereology. Stereological point-counting confirmed our observations of an age-dependent increase in Aβ plaque load (Kruskal–Wallis test, *P* < 0.0001) (Figure 1B). The largest increase in % Aβ plaque load occurred between 12 and 15 months of age, increasing from 2.2 to 4.5% of the hippocampus now covered by Aβ plaques. The maximal % Aβ plaque load was 6% at 21 and 24 months. A sigmoidal trajectory in % Aβ plaque load was determined using non-linear regression (*R*^2^ = 0.86, Figure 1B), which is consistent with observations in the neocortex in both AD (Serrano-Pozo et al., 2011; Jack et al., 2013) and APP_swe_/PS1_ΔE9_ Tg mice (Babcock et al., 2015). Statistically significant increases in % Aβ plaque load were observed from 15 months of age, compared to 3-month-old mice (Figure 1B, asterisks). No significant differences in hippocampal volume were observed in Tg mice between 3 to 24 months of age (Figure 1C). As expected, no Aβ plaques were observed in WT mice at any age (data not shown).

### Microglia Accumulate at Aβ Plaques in the Hippocampus of APP_swe_/PS1_ΔE9_ Tg Mice

**FIGURE 2:**
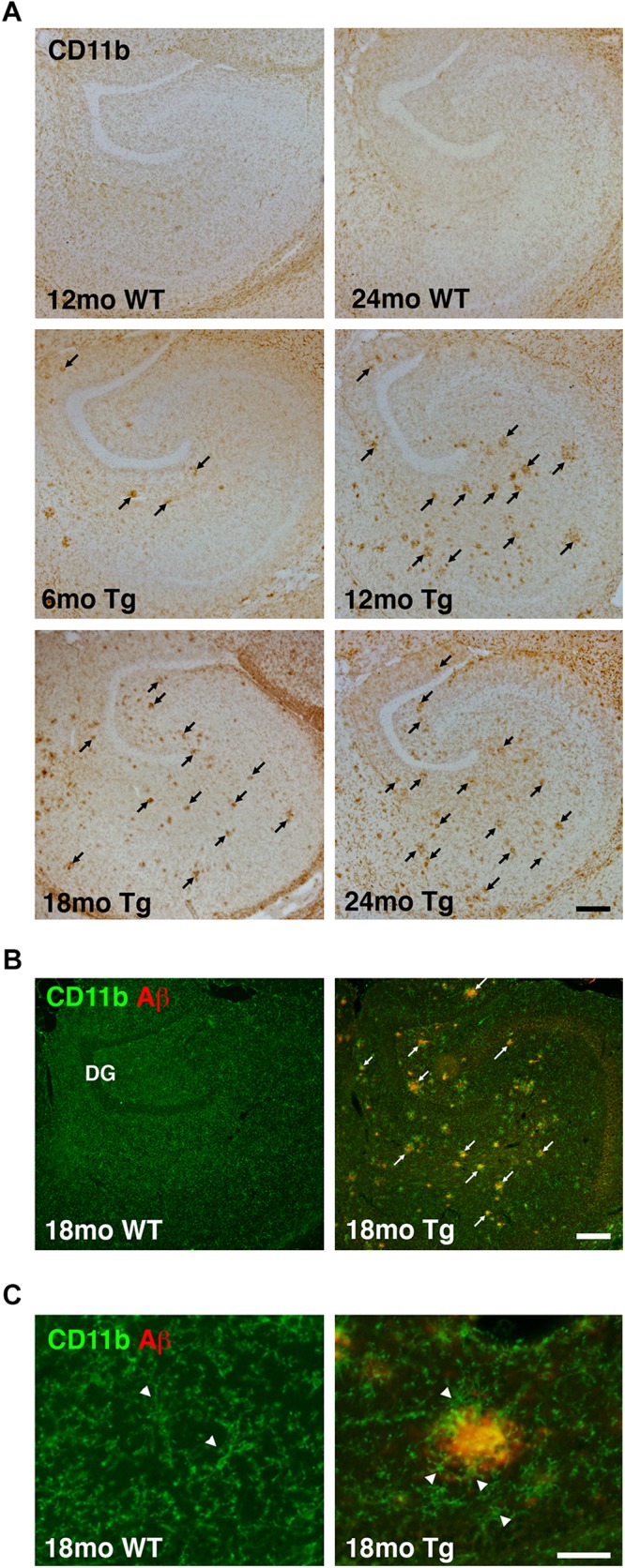
Altered microglial distribution in the hippocampus of APP_swe_/PS1_ΔE9_ Tg mice. **(A)** Low magnification panels show changes in CD11b immunoreactivity in the hippocampus of 6-, 12-, 18-, and 24-month-old Tg mice. The hippocampus from 12- and 24-month-old WT mice is shown for comparison. **(B,C)** Combined immunofluorescence staining for CD11b and Aβ shows increased CD11b immunoreactivity near Aβ plaques in low magnification images from 18-month-old Tg mice **(B)**. No plaques are observed in age-matched WT mice **(B)**. Arrowheads in **(C)** point to CD11b^+^ microglia. Microglial cells are clustered around Aβ plaques in Tg mice, but not in WT mice. The photomicrographs in **(C)** were both obtained in the dentate molecular layer. DG, dentate gyrus. Scale bars: 200 μm **(A,B)**, 500 μm **(C)**.

We next analyzed changes in microglial reactivity. The IHC staining for the microglial surface β-integrin CD11b was homogeneously distributed in the hippocampus of WT mice, reflecting an even distribution of microglia even in 24-month-old mice (Figures 2A–C). In contrast, changes in microglial CD11b immunoreactivity were readily apparent in the hippocampus in APP_swe_/PS1_ΔE9_ Tg mice (Figure 2A). Aggregates of CD11b^+^ microglia were clearly visible in the hippocampus of Tg mice at 6 months and were more numerous at 12, 18, and 24 months (Figure 2A). These aggregates seemed to coincide temporally with the appearance of Aβ plaques (Figure 1A), initially forming in areas innervated by the perforant pathway before becoming prominent in the dentate hilus and other hippocampal regions in aged APP_swe_/PS1_ΔE9_ Tg mice (Figure 2A). In double-stained sections, CD11b aggregates in these regions overlapped with Aβ plaques (Figure 2B), with microglia clustering around Aβ plaques (Figure 2C), as also reported for microglia in the neocortex of the same mice (Babcock et al., 2015).

### IGF-1 mRNA Levels Increase With Age in the Hippocampus of APP_swe_/PS1_ΔE9_ Tg Mice

**FIGURE 3:**
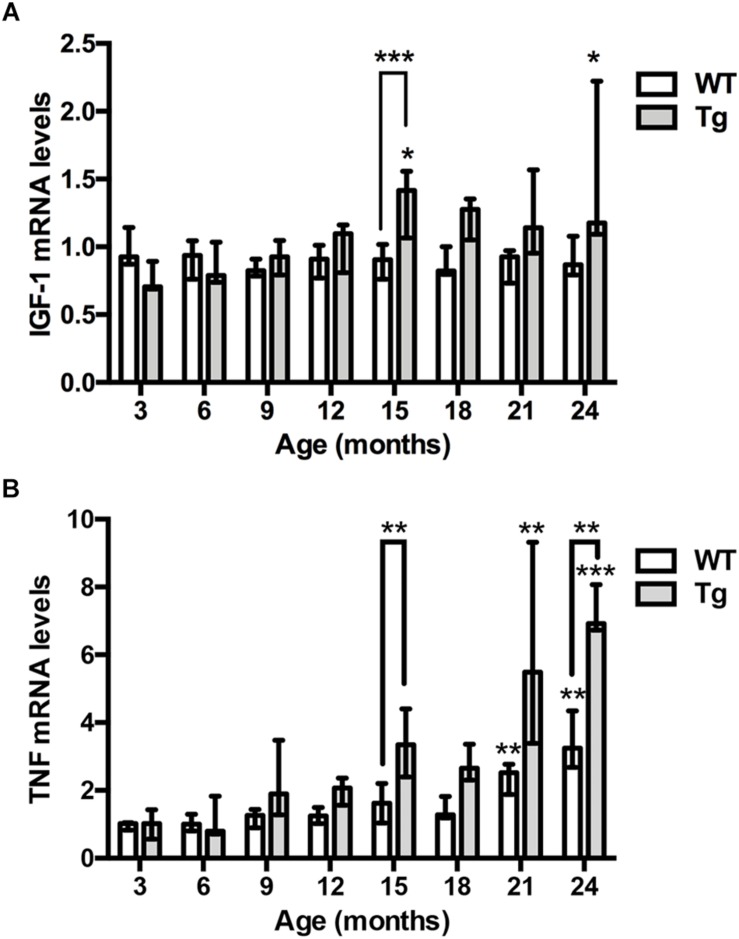
IGF-1 and TNF mRNA levels are elevated in the hippocampus of aging APP_swe_/PS1_ΔE9_ Tg mice. **(A)** Quantitative PCR analysis shows significantly increased hippocampal IGF-1 mRNA levels with age in Tg mice (gray bars), but not WT mice (white bars). Bars represent medians with 25 and 75% quartiles (*n* = 6–10 per group, except for *n* = 4 for 18-month-old Tg mice). **(B)** Quantitative PCR analysis shows significantly increased TNF mRNA levels with age in the hippocampus of both Tg mice (gray bars) and WT mice (white bars). Bars represent medians with 25 and 75% quartiles (*n* = 6–10 per group). ^*^p < 0.05, ^∗∗^*p* < 0.01, ^∗∗∗^*p* < 0.001, ^∗∗∗^*p* < 0.0001, based on Kruskal–Wallis test followed by Dunn’s multiple comparison test.

To assess the expression of IGF-1 mRNA relative to age and genotype, we examined IGF-1 mRNA levels in contralateral hippocampi from the 3- to 24-month-old APP_swe_/PS1_ΔE9_ Tg mice used for Aβ plaque load estimation, and from age-matched WT mice. We detected significant, but less than 2-fold increases in IGF-1 mRNA levels in 15- and 24-month-old Tg mice compared to young, 3-month-old Tg mice (Kruskal–Wallis test, *P* < 0.05, both age groups) (Figure 3A). In the APP_swe_/PS1_ΔE9_ Tg mice the 15-month-old mice showed significantly higher IGF-1 mRNA levels compared to age-matched WT mice (Dunn’s test, *P* < 0.05, both age groups) (Figure 3A). In accordance with these results, IGF-1 mRNA levels were significantly correlated with % Aβ plaque load (*r* = 0.56, *P* < 0.0001) and age (*r* = 0.54, *P* < 0.001) in Tg mice, while no correlation to age was observed in the WT mice (Table 1). In conclusion, the aging APP_swe_/PS1_ΔE9_ Tg mice showed a significant increase in IGF-1 mRNA levels, which correlated to the age-dependent increase in % Aβ plaque load in these mice.

**TABLE 1:** Pearson correlations between cytokine mRNA levels, and age and % Aβ plaque load, in the hippocampus of 3–24-month-old WT and APP_swe_/PS1_ΔE9_ Tg mice.

**Genotype and variable / Cytokine mRNA levels**	**WT**	**APP_swe_/PS1**_Δ_**_E9_**	
		
	**Age**	**Age**	**% Aβ plaque load**
TNF mRNA levels	r 0.68, *P* < 0.0001	r 0.73, *P* < 0.0001	r 0.63, *P* < 0.0001
IGF-1 mRNA levels	r 0.00, ns	r 0.54, *P* < 0.0001	r 0.56, *P* < 0.0001

### TNF mRNA Levels Increase With Age in the Hippocampus of APP_swe_/PS1_ΔE9_ Tg and WT Mice

Next, we assessed whether and how hippocampal TNF mRNA levels might change relative to age and genotype (Figure 3B). We detected significant, up to 3–4-fold increases in TNF mRNA levels in 21- and 24-month-old WT mice compared to young, 3-month-old WT mice (Kruskal–Wallis test, *P* < 0.01, both age groups) (Figure 3B). In APP_swe_/PS1_ΔE9_ Tg mice, TNF mRNA levels showed a 7–8-fold increase at 21 and 24 months, compared to 3-month-old Tg mice (Kruskal–Wallis test, *P* < 0.01 and *P* < 0.001, respectively) (Figure 3B). Due to the age-associated increases in TNF mRNA levels also in the WT mice, TNF mRNA levels were maximally 2.5-fold higher in Tg mice compared to WT mice at a given age (Figure 3B). In the APP_swe_/PS1_ΔE9_ Tg mice the 15- and 24-month-old mice showed significantly higher TNF mRNA levels compared to age-matched WT mice (Dunn’s test, *P* < 0.01, both age groups) (Figure 3B). As suggested by these results, TNF mRNA levels across all mouse groups correlated to both the % Aβ plaque load (*r* = 0.63, *P* < 0.0001) as well as age (*r* = 0.73, *P* < 0.0001) in APP_swe_/PS1_ΔE9_ Tg mice (Table 1), and to age in WT mice (*r* = 0.68, *P* < 0.0001). Having previously shown rare TNF mRNA^+^ cells in the neocortex of the same mice (Babcock et al., 2015), we also analyzed the expression of TNF mRNA in the hippocampus. TNF mRNA^+^ cells were scarce even in the 24-month-old WT and APP_swe_/PS1_ΔE9_ Tg mice (Supplementary Figure S1). TNF mRNA^+^ cells were observed in all hippocampal subregions, regardless of age and genotype (data not shown).

### Cellular and Regional Expression of IGF-1 mRNA in the Hippocampus of APP_swe_/PS1_ΔE9_ Tg Mice

**FIGURE 4:**
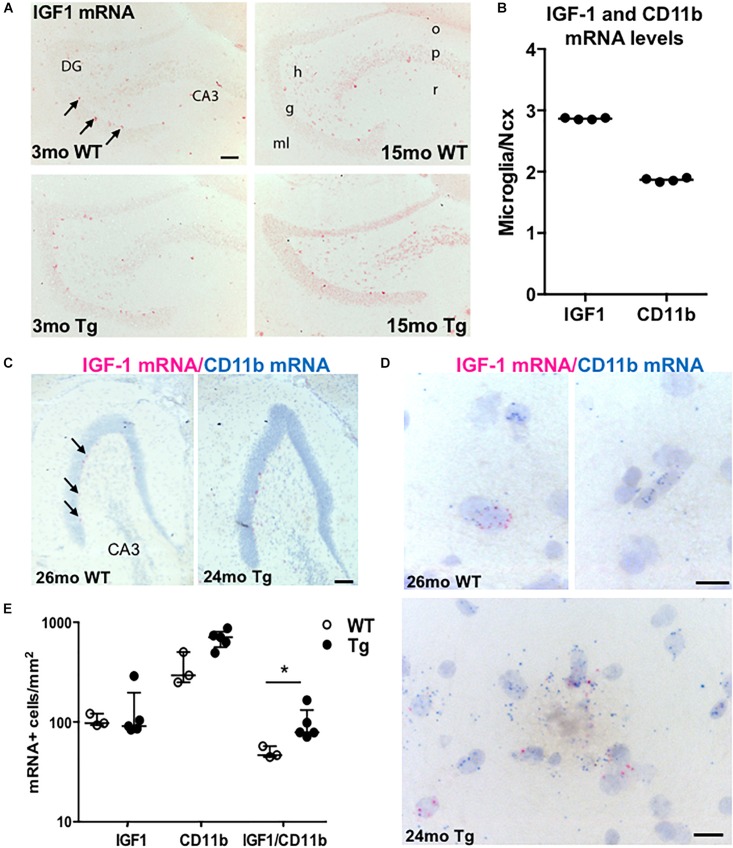
IGF-1 mRNA is expressed in microglia as well as neurons in APP_swe_/PS1_ΔE9_ Tg mice. **(A)** *ISH* showing IGF-1 mRNA^+^ cells in the hippocampus of 3- and 15-month-old WT and Tg mice. *ISH* was performed with AP-conjugated probes. The only clearly visible cells are sgz cells bordering the granular cell layer (arrows) and scattered neurons in the dentate hilus and stratum radiatum of CA3. **(B)** Scatter-plot showing IGF-1 and CD11b mRNA levels in primary microglia relative to Ncx samples from 3-month-old C57BL/6 mice. Each data point represents one microglial culture from the same experiment. The horizontal bar represents the median. Both genes are more abundantly expressed in primary microglia than in whole Ncx tissue. **(C,D)** View-RNA double ISH for IGF-1 mRNA (red) and CD11b mRNA (blue) in approx. two-year-old WT and Tg mice. Arrows in **(C)** point at sgz cells expressing IGF-1 mRNA, as also observed in **(A)**. High magnifications in **(D)** show single cells expressing IGF-1 mRNA and/or CD11b mRNA in a WT mouse (top panels) and in a Tg mouse (bottom panel). Note the amorphous material in the bottom panel reflecting the presence of an amyloid plaque. **(E)** Scatter-plot showing the number of IGF-1 mRNA^+^, CD11b mRNA^+^, and IGF mRNA-expressing CD11b mRNA^+^ microglia in WT and Tg mice. Bars represent medians **(B,E)** with 25 and 75% quartiles **(E)**. CA3, regio inferior hippocampus; DG, dentate gyrus; h, hilus; g, granule cell layer; ml, molecular layer; o, stratum oriens; p, pyramidal cell layer; and r, stratum radiatum. ^*^p < 0.05, Mann–Whitney, unpaired, and two-tailed. Scale bars: 100 μm **(A,C)**, 20 μm **(D)**.

To know the distribution and the source of the IGF-1 mRNA-expressing cells, we next hybridized sections parallel to those used for Aβ plaque load estimation using an AP-conjugated probe specific for IGF-1 mRNA. We observed numerous IGF-1 mRNA^+^ neuronal-like cells throughout the hippocampus of the adult and aging APP_swe_/PS1_ΔE9_ Tg and WT mice, with predilection to the sgz, defining the border between the granule cell layer and the dentate hilus (Figure 4A). IGF-1 mRNA^+^ neuronal-like cells were also relatively abundant in the stratum radiatum of CA3 and CA1 (data not shown). Besides the neuronal-like cells, a more widespread punctuate staining was observed in the neural tissue, corresponding to a low baseline expression of IGF-1 mRNA by both neurons and glia (data not shown). Despite of the increased microglial reactivity in the Tg mice (Figures 2A,B), we observed no clear differences in *ISH* signal between Tg and WT mice using the AP-conjugated probe (Figure 4A). Therefore the expression level of IGF-1 mRNA was also analyzed in primary murine microglia from C57BL/6 mice by use of qPCR. The mRNA levels of IGF-1 and CD11b were determined relative to the expression level in neocortex of 3-month-old C57BL/6 mice. IGF-1 and CD11b mRNA levels were respectively, 3- and 2-fold higher in primary microglia compared to whole neocortex tissue (Figure 4B).

Next, we hybridized tissues from P5 mouse pups, which are known to contain high numbers of IGF-1 mRNA-expressing microglia (Hammond et al., 2019), showing a predilection to locate in the developing corpus callosum (Wlodarczyk et al., 2017), which was confirmed by our *ISH* (Supplementary Figure S2A). With their round nuclei these cells ressembled amoeboid microglia (Dalmau et al., 2003). IGF-1 mRNA was also expressed in amoeboid-like cells in the developing angular bundle (Supplementary Figure S2A), and in cells with a neuronal morphology, which were observed throughout the hippocampus, including the stratum radiatum of CA3 and CA1 and the sgz in the dentate gyrus (Supplementary Figure S2A). Additionally, the cerebellar Purkinje cells expressed high levels IGF-1 mRNA (data not shown). Compared to the hybridized sections, sections pre-treated with RNAse A prior to hybridization or hybridized with an excess of unlabelled probe (competition control) were devoid of signal (Supplementary Figure S2B).

In combination, the detection of high levels of IGF-1 mRNA in primary microglia and the *in situ* detection of IGF-1 mRNA in amoeboid-like microglia in developing white matter raised the possibility that we might also be able to detect IGF-1 mRNA in activated microglia in the aged APP_swe_/PS1_ΔE9_ Tg mice.

### More Microglia Express IGF-1 mRNA in the Dentate Molecular Layer in Aged APP_swe_/PS1_ΔE9_ Tg Than WT Mice

We then investigated if the increased levels of IGF-1 mRNA in the hippocampus of the aged APP_swe_/PS1_ΔE9_ Tg mice might be ascribed to microglia expressing IGF-1 mRNA. We therefore tried to co-localize IGF-1 mRNA to CD11b mRNA^+^ microglia in aged Tg mice and WT mice (*n* = 2–3/group) using the sensitive View-RNA *ISH* technique (Grebing et al., 2016). As expected, neuronal-like IGF-1 mRNA^+^ sgz cells were readily observed in overview images of the dentate gyrus (Figure 4C). High magnification images, showed co-localization of IGF-1 mRNA to CD11b mRNA^+^ microglia in both WT and Tg mice (Figure 4D). Quantitative analysis showed that the density of IGF-1 mRNA^+^ CD11b mRNA^+^ microglia was higher in hippocampi from Tg compared to WT mice (Figure 4E and Supplementary Table S2). Since the number of CD11b mRNA^+^ microglia was also higher (Figure 4E), the percentage of IGF-1 mRNA^+^ microglia was comparable in Tg and WT mice (15.9% and 14.5%, respectively). Taken together, this suggest that the increased levels of IGF-1 mRNA in the aged APP_swe_/PS1_ΔE9_ Tg mice can be attributed to an increased number of IGF-1 mRNA^+^ microglia in these mice.

### IGF-1 Immunoreactivity Is Abundant in Neurons and Can Be Detected in Microglia in Aged APP_swe_/PS1_ΔE9_ Tg Mice

**FIGURE 5:**
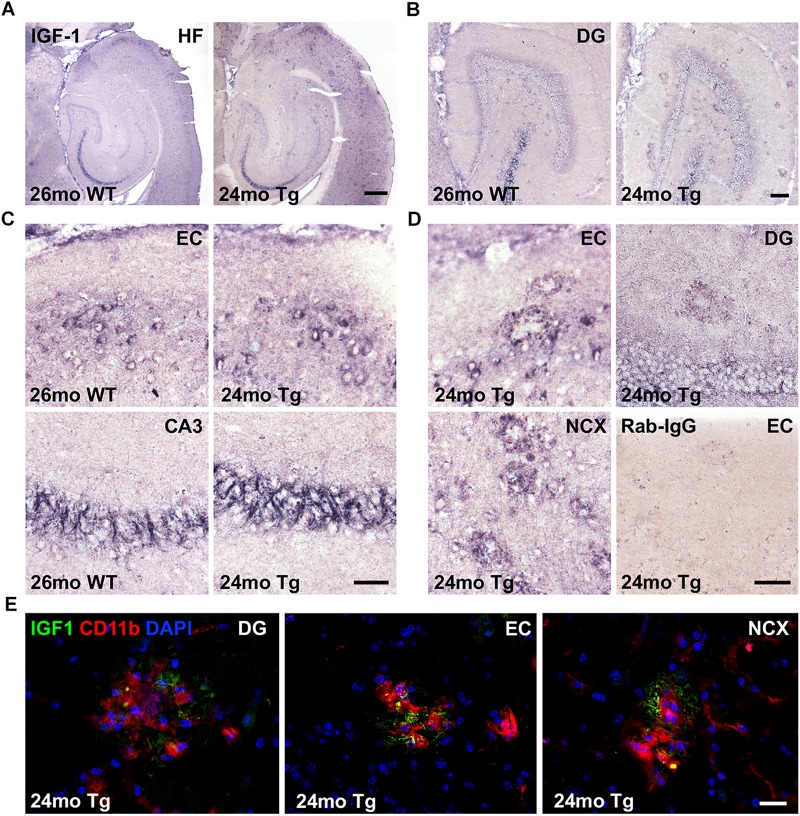
IGF-1 is expressed in neurons and a subset of microglia in APP_swe_/PS1_ΔE9_ Tg mice. **(A–C)** IHC of sections from 24-month-old APP_swe_/PS1_ΔE9_ Tg and 26-month-old WT mice showing IGF-1 immunoreactivity in the hippocampal formation **(A)** and dentate gyrus **(B)**, with higher magnification of neurons in the entorhinal cortex and fiber-like structures in CA3 **(C)**. **(D)** High magnifications from Tg mice showing amyloid plaque-associated punctuate IGF-1 immunoreactivity in dentate gyrus, entorhinal cortex, and neocortex. Substitution control performed with inert rabbit IgG showed no staining. CA3, regio inferior hippocampus; DG, dentate gyrus; EC, entorhinal cortex; HF, hippocampal formation; and NCX, neocortex. **(E)** Double immunofluorescence for IGF-1 (green) and CD11b (red), and nuclear staining with DAPI (blue). Co-localization of IGF-1 to CD11b^+^ microglia is visualized by the punctuate yellow staining, whereas the IGF-1^+^ fiber-like structures in the plaques remain green. Scale-bars: 400 μm **(A)**, 100 μm **(B)**, 50 μm **(C,D)**, and 20 μm **(E)**.

To examine whether Aβ plaque-associated microglia produce IGF-1 protein in the aged APP_swe_/PS1_ΔE9_ Tg mice, we next stained sections parallel to those used for co-expression analysis for IGF-1 mRNA and CD11b mRNA by IHC for IGF-1. IGF-1 immunoreactivity was abundant in subsets of neurons in the hippocampal formation (Figures 5A–C), especially in layer II of the entorhinal cortex (Figure 5C), and in fiber-like structures in the CA3 pyramidal cell layer (Figures 5A–C). IGF-1 immunoreactivity was also observed in scattered neurons in the dentate hilus (Figure 5B) and in the neocortex (data not shown), and it was abundant in the Purkinje cells in the cerebellum (Supplementary Figure 3A). In addition to the neuronal IGF-1 immunoreactivity, an Aβ plaque-associated punctuate IGF-1 immunoreactivity was observed in the Tg mice (Figure 5D). This type of IGF-1 immunoreactivity was most abundant in the entorhinal cortex and in neocortex, but could also be seen in the dentate gyrus (Figure 5D). Frequently, IGF-1 immunoreactivity was also observed on fiber-like structures in association with the plaques (data not shown). Importantly, substitution of the primary antibody with inert rabbit IgG abolished all staining (Figure 5D and Supplementary Figure S3B).

Next, to clarify whether the punctuate IGF-1 staining might co-localize to microglia we performed double-immunofluorescence staining for IGF-1 and CD11b. Punctuate IGF-1 immunofluorescence signal could be detected in a subset of CD11b^+^ microglia associated with the Aβ plaques (Figure 5E, orthogonal views in Supplementary Figures S4, S5, S6) and fiber-like structures in the plaques (Figure 5E). As described above, IGF-1 immunofluorescence was abundant in entorhinal layer II neurons, fibers in the CA3 pyramidal cell layer as well as in cerebellar Purkinje cells (Supplementary Figure S7). Substitution control revealed unspecific binding of the secondary AlexaFluor568 goat-anti rat IgG to the vasculature in our specimens (Supplementary Figure S7). However, due to the morphological characteristics of the microglia this did, however, not interfere with interpretation of the double-immunofluorescence staining. In conclusion, IGF-1 is abundantly expressed in subsets of neurons in both genotypes and in a subset of Aβ plaques-associated microglia in aged APP_swe_/PS1_ΔE9_ Tg mice.

### IGF-1 mRNA Expression in Microglial BV2 Cells Is Unaffected by Addition of Aβ_42_

**FIGURE 6:**
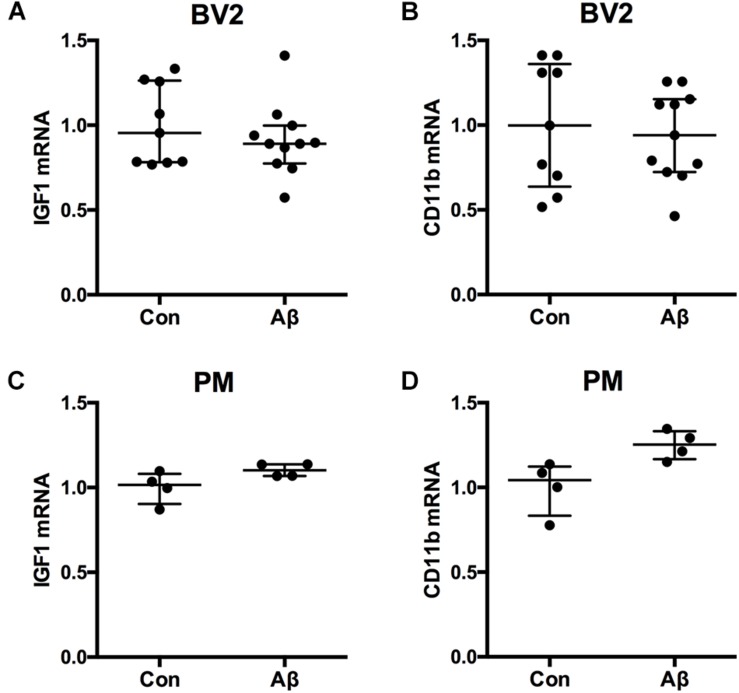
IGF-1 mRNA is unchanged in BV2 cells and primary microglia after Aβ_42_ exposure. The BV2 cell line and primary microglia from newborn mice were stimulated 24 h with 1 μM Aβ_42_ whereafter, RNA was isolated, reverse transcribed and used for qPCR for IGF-1 mRNA **(A,C)** and CD11b mRNA **(B,D)**. Levels of IGF-1 mRNA **(A,C)** and CD11b mRNA **(B,D)** were unchanged after Aβ_42_ exposure. Each dot represents 1 experiment. Bars indicate the medians and the error bars the 25 and 75% quartiles.

Microglial content of Aβ was previously shown to be functionally correlated to microglial cytokine expression *in vivo* (Babcock et al., 2015). To clarify whether Aβ might impact microglial expression of IGF-1 mRNA, we examined the effect of Aβ_42_ on the IGF-1 mRNA expression in microglial BV2 cells. For the stimulation was used Aβ_42_ which is more prevalent than Aβ_40_ in the hippocampus and neocortex of aging Tg mice (Babcock et al., 2015; von Linstow et al., 2017). As a reference was included CD11b mRNA. Aβ_42_ exposure of microglial BV2 cells for 24 h had no impact on neither IGF-1 nor CD11b mRNA expression (Figures 6A,B). In support, Aβ_42_ exposure for 24 h of primary microglia from newborn C57BL/6 mice did not impact IGF-1 or CD11b mRNA expression either (Figures 6C,D).

### Cell Proliferation in the sgz Decreases With Age

**FIGURE 7:**
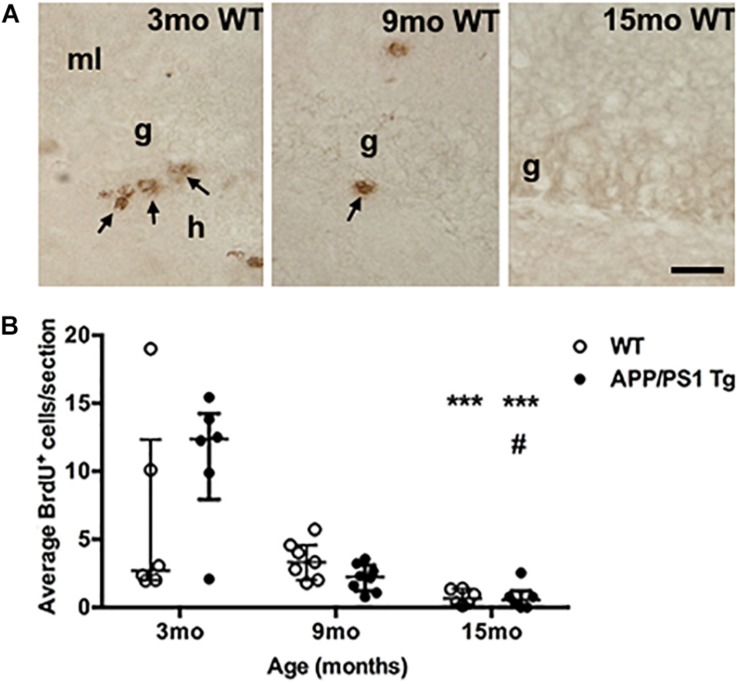
Cell proliferation in the subgranular zone of both genotypes decreases with age. **(A)** Immunohistochemical staining of proliferating BrdU^+^ cells (arrows) in the subgranular zone of 3-, 9-, and 15-month-old WT mice. The BrdU-staining was performed on sections from post-fixed, isolated hippocampi. In general, fewer BrdU^+^ cells were observed in the subgranular zone at 9 months of age, and at 15 months, almost no BrdU^+^ cells were observed in either Tg (not shown), or WT mice. g, granular cell layer; h, hilus; and ml, molecular layer. Scale bar: 20 μm. **(B)** Scatter-plot showing the average number of BrdU^+^ cells in the sgz 3-, 9-, and 15-month-old WT and APP_swe_/PS1_ΔE9_ Tg mice. Despite of a large variation in the number of BrdU^+^ cells in 3-month-old mice (see text for details) there was a significant age-dependent reduction in BrdU^+^ cells in both APP_swe_/PS1_ΔE9_ Tg and WT mice by 15 months of age. Data are expressed as the mean number of BrdU^+^ cells per section and are shown as mean ± sem for each group (*n* = 6–8 per group). No significant differences were observed between WT and Tg mice. #*p* < 0.05 (vs. 9-month-old Tg mice), ^∗∗∗^*p* < 0.0001 (vs. 3-month-old mice).

We finally asked whether the increased Aβ plaque load in the female APP_swe_/PS1_ΔE9_ Tg mice might contribute to age-related differences in cellular proliferation in the dentate gyrus. We therefore quantified proliferating (BrdU^+^) cells in the sgz of the dentate gyrus where neural precursors are located (Zhao et al., 2008). To assess changes relevant to our assessment of the Aβ plaque-induced changes in cytokine mRNA levels, we included groups of 3-, 9-, and 15-month-old Tg and WT mice. At 3 months of age, we observed clusters of BrdU^+^ cells in the sgz in both Tg and WT mice (Figure 7A, data shown for WT mice). These clearly decreased at 9 months of age, and at 15 months almost no BrdU^+^ cells were observed in either Tg or WT mice (Figure 7A). Quantification showed a significant age-dependent reduction in BrdU^+^ cells in both Tg and WT mice (Kruskal–Wallis test, *P* < 0.001, both genotypes) (Figure 7B). Statistically significant decreases in number of BrdU^+^ were observed at 15 months of age, compared to 3-month-old mice in both genotypes (Figure 7B, asterisks). We did not observe any significant differences between WT and Tg mice in any age group. Four out of 6 WT mice and one out of 6 Tg belonging to group of 3-mo-old mice showed very low cell numbers, which might be attributed to suboptimal BrdU-staining. Finally, hippocampal mRNA levels of brain derived neurotrophic factor (BDNF), which is known to promote neurogenesis (Lichtenwalner and Parent, 2006), remained constant across ages in both Tg and WT mice (Supplementary Figure S8).

## Discussion

The main results of this study showed that hippocampal IGF-1 mRNA levels are increased close to two-fold in aged APP_swe_/PS1_ΔE9_ Tg mice, that approx. 15% of microglia in the molecular layer of the dentate gyrus in aged Tg as well as WT mice express IGF-1 mRNA, and that a subset of microglia in aged Tg mice express IGF-1 protein. Since microglial density was approx. two-fold higher in Tg mice compared to WT mice, the Tg mice also harbored two-fold more IGF-1 mRNA-expressing microglia. Thus, the increased hippocampal IGF-1 mRNA levels may be ascribed to an increased number of IGF-1-expressing microglia. Still, the most predominant cell types expressing IGF-1 mRNA, were neurons, including neuroblast-like cells in the sgz. Neurogenesis, given by the number of proliferating cells in the sgz, declined with age in both genotypes, regardless of the genotype-associated differences in hippocampal IGF-1 mRNA levels. The strength, limitations and perspectives of these findings, and of our results on the genotype- and age-associated changes in hippocampal TNF mRNA levels, are discussed below.

We found that % Aβ plaque load increased with age in the hippocampus of APP_swe_/PS1_ΔE9_ Tg mice and reached a maximum in 21-month-old mice. The resulting sigmoidal curve corresponds to observations in the neocortex in AD patients (Serrano-Pozo et al., 2011; Jack et al., 2013) and the same APP_swe_/PS1_ΔE9_ Tg mice (Babcock et al., 2015). This apparent maximum limit for hippocampal Aβ-load might explain why Aβ plaque load is not well correlated with cognitive symptoms in AD patients (Nelson et al., 2012; Jack et al., 2013). Cognitive symptoms appear to be a consequence of neurodegeneration, whereas Aβ deposition is an “upstream” event (Jack et al., 2013). Since APP_swe_/PS1_ΔE9_ Tg mice are not genetically modified to produce neurofibrillary tangles, which may trigger neuronal death (Braak and Braak, 1995; Eckermann et al., 2007), we did not expect to see marked changes in hippocampal volume. Although a modest neuronal loss around plaques has been reported in aged APP_swe_/PS1_ΔE9_ Tg mice (Radde et al., 2006, Rupp et al., 2011), no global loss of hippocampal CA1 neurons was observed in APP_swe_/PS1_ΔE9_ Tg mice at 12 months of age (West et al., 2009). We observed no age- or genotype-associated changes in neocortex volume in our former study (Babcock et al., 2015).

We found that a subset of microglia in aged APP_swe_/PS1_ΔE9_ Tg mice synthesized IGF-1 mRNA and IGF-1 protein. These findings are in line with reports of microglial expression of IGF-1 mRNA after perforant pathway deafferentation in rats (Guthrie et al., 1995), overlapping with zones where we found Aβ plaque load to be prominent. Deposition of Aβ can induce modest damage to axons (Liu et al., 2008, Nikolajsen et al., 2011), dendrites and neuronal somata (Radde et al., 2006, Rupp et al., 2011). In addition, Aβ plaques were also prominent in the entorhinal cortex giving rise to the perforant pathway. Microglia may sense degenerating neurons and Aβ via the same cell surface receptors (Babcock et al., 2006; Jana et al., 2008), and Aβ, potentially in combination with Aβ plaque-associated neuronal damage, may provoke a response similar to perforant pathway axonal degeneration. The microglial population increases two- to four-fold in the outer part of the dentate molecular layer in response to perforant pathway axonal degeneration in mice (Dissing-Olesen et al., 2007, Wirenfeldt et al., 2007). In the present study, we found that microglial density was two-fold higher in the molecular layer in APP_swe_/PS1_ΔE9_ Tg mice compared to WT mice, and that the Tg mice also harbored two-fold more IGF-1 mRNA-expressing microglia. Whether individual microglia express more IGF-1 mRNA per cell in aged Tg compared to WT mice requires more comprehensive studies, also addressing differences in IGF-1 mRNA expression between the different IGF-1 mRNA-expressing cell types in the dentate gyrus and hippocampus proper, as well as the effect on aging. Microglia isolated from the brains of aged C57BL/6 mice were previously reported to express higher IGF-1 mRNA levels than microglia from young mice (Hickman et al., 2013). The reason for the absence of effect of Aβ42 on the IGF-1 mRNA expression by the BV2 cells and the primary microglia could be that these cells already express high levels of IGF-1 mRNA, which the published data from the P5 pubs (Wlodarczyk et al., 2017; Hammond et al., 2019) and our own *ISH* data indicate. Another reason could be that a combination of Aβ42 and Aβ40 might be more efficient in stimulating microglial IGF-1 mRNA expression than Aβ42.

IGF-1 has previously been shown to modulate hippocampal neurogenesis (Aberg et al., 2000, 2003; Anderson et al., 2002), and the cell type showing the most intense *ISH* signal for IGF-1 mRNA, across ages and genotypes, were the neuroblast-like cells in the sgz. With their characteristic distribution and morphology these cells resembled the doublecortin-expressing neuroblasts which we showed are reduced in 18-month-, but not 9- or 3-month-old male APP_swe_/PS1_ΔE9_ Tg mice (Olesen et al., 2017). Enhancing microglial expression of IGF-1 in 9- to 10-month-old APP_*K95N*,__*M591L*_/PS1_ΔE9_ Tg mice by glatiramer acetate treatment was previously suggested as a mechanism to enhance neurogenesis and counteract learning deficits (Butovsky et al., 2006). In comparison, in our present study the elevated hippocampal IGF-1 mRNA levels in the aged APP_swe_/PS1_ΔE9_ Tg mice did not influence neurogenesis, which was similarly low in the 15-month-old female and the 18-month-old male Tg and littermate WT mice (the present study and Olesen et al., 2017, respectively). Importantly, although the changes we observed were less than two-fold, IGF-1 mRNA was expressed at relatively high levels in the hippocampus of both WT and APP_swe_/PS1_ΔE9_ Tg mice, typically detected by qPCR at 8–10 cycle thresholds before TNF. The fact that baseline-level of IGF-1 mRNA is relatively high in the hippocampus, and that several cellular sources, including neurons, microglia, as well as sgz cells, produce IGF-1 mRNA, calls for caution in the interpretation of the contribution of microglial versus non-microglial cells as sources of the functionally important IGF-1. Additionally, increased expression of IGF-1 mRNA may be counteractive, since Aβ can reduce neuronal sensitivity to IGF-1 by decreasing neuronal IGF-1Rs (Giuffrida et al., 2012). Reduced IGF-1 sensitivity in APP_swe_/PS1_ΔE9_ Tg mice has been observed (Zhang et al., 2013), as has deregulated IGF-1 signaling (Zemva and Schubert, 2014).

We also observed increased TNF mRNA levels in APP_swe_/PS1_ΔE9_ as well as WT mice with age. Increased hippocampal TNF mRNA and protein have previously been reported in aged APP_swe_/PS1_ΔE9_ Tg mice (Francois et al., 2014; Minogue et al., 2014). Though the cell source of TNF mRNA in hippocampus was not determined by double-ISH, as done for IGF-1 mRNA and CD11b mRNA, we have previously shown that neocortical microglia produce TNF in aging WT and APP_swe_/PS1_ΔE9_ Tg mice (Babcock et al., 2015). Also, the age-related increase in hippocampal TNF mRNA fits well with the reported increase in TNF mRNA expression in microglia from aged C57BL/6 mice (Hickman et al., 2013). TNF might also be produced by infiltrating macrophages (Clausen et al., 2008; Lambertsen et al., 2009) as immigrating cells can be detected in the hippocampus of chimeric APP_swe_/PS1_ΔE9_ Tg mice, though greatly outnumbered by microglia (Babcock et al., 2015). As expected based on our quantitative studies of TNF mRNA^+^ cells in the neocortex of the same APP_swe_/PS1_ΔE9_ Tg mice (Babcock et al., 2015), we found very low numbers of TNF mRNA^+^ cells, distributed sporadically throughout the hippocampus, though absent from the neurogenic niche in the sgz in both genotypes. This suggests that fold-increases in TNF mRNA measured at the whole hippocampus level by qPCR may reflect individual cells expressing high levels of TNF mRNA.

In this study, we used IHC staining for CD11b for visualization of microglial activation. Changes in CD11b immunoreactivity could be observed in all hippocampal subregions of APP_swe_/PS1_ΔE9_ Tg mice with CD11b^+^ cellular aggregates coinciding with Aβ plaques, as previously reported (Bolmont et al., 2008; Minogue et al., 2014, Babcock et al., 2015), and with Aβ plaques and microglial aggregates in the dentate hilus, in close association with the sgz. Although microglial phagocytosis of apoptotic new-born neurons is maintained during aging and acute inflammation (Sierra et al., 2010), the additional challenge of being exposed to and to take up Aβ could change how efficiently microglia can support this key physiological process during neurogenesis. A significant subpopulation of microglia in APP_swe_/PS1_ΔE9_ Tg mice has a high Aβ load (Babcock et al., 2015), and having to clear Aβ interfered with phagocytic uptake of beads in the hippocampus of another Tg mouse model (Krabbe et al., 2013). Interestingly, microglial capacity for phagocytosis appears to be related to the cytokine profile (Babcock et al., 2015), however, while we know that the microglial subset expressing TNF is poor in taking up Aβ, we have no data on whether microglial expression of IGF-1 impacts their uptake of Aβ. Recently, we showed by the use of proteomics that CD11b^+^ myeloid cells (mainly microglia) isolated from 24-month-old APP_swe_/PS1_ΔE9_ Tg mice have lower expression of IGF binding protein 2 (IGFBP2) compared to CD11b^+^ myeloid cells from 24-month-old WT mice (Thygesen et al., 2018). IGFBP2 is also expressed in microglia in active lesions in multiple sclerosis cases (Chesik et al., 2004). Secretion of IGFBP2 is important for transport of IGF-1 to its receptors. While we did not identify IGF-I as being differentially regulated in our proteomics study (Thygesen et al., 2018), likely due to the technical limitation on low molecular protein identification by mass spectrometry (Nilsson et al., 2010), we in the present study found IGF-1 co-localized to a subset of plaques-associated CD11b^+^ microglia. Taking into consideration that the double-immunofluorescence staining was a compromise regarding fixation protocols, to allow simultaneous detection of CD11b and IGF-1, the frequency of IGF-1^+^ microglia is likely underestimated. Indeed, IGF-1 mRNA was shown to be among the significantly upregulated genes in microglia from 6-month-old male 5XFAD mice in recent single-cell RNA-sequencing studies, classifying the IGF-1 mRNA^+^ microglia as homeostatic microglia (Keren-Shaul et al., 2017).

Decreased sgz cell proliferation in normal aging is well-documented in both humans (Spalding et al., 2013) and rodents (Kuhn et al., 1996; Demars et al., 2010; Valero et al., 2011; Villeda et al., 2011; Hamilton and Holscher, 2012; Olesen et al., 2017). In comparison, in AD, sgz proliferation has been reported to be both increased (Jin et al., 2004) and decreased (Ziabreva et al., 2006; Crews et al., 2010), similar to inconsistencies described in different mouse models of AD (Chuang, 2010). Several studies reported more pronounced decreases in APP_swe_/PS1_ΔE9_ Tg than WT mice (Butovsky et al., 2006; Demars et al., 2010; Valero et al., 2011; Hamilton and Holscher, 2012). We recently reported that sgz cell proliferation decreased to the same extent in APP_swe_/PS1_ΔE9_ Tg and littermate WT mice at 18 months of age, while we did observe a larger reduction in the number of doublecortin immunoreactive neuroblasts in the APP_swe_/PS1_ΔE9_ Tg mice (Olesen et al., 2017).

## Conclusion

In conclusion, the results suggest that the increased IGF-1 mRNA levels observed in aged APP_swe_/PS1_ΔE9_ Tg mice can be ascribed to a larger number of IGF-1 mRNA-expressing microglia, and additionally that IGF-1 mRNA is translated into IGF-1 protein in a subset of Aβ-plaque-associated microglia. The finding that microglia retain the capacity to produce IGF-1 in the aged APP_swe_/PS1_ΔE9_ Tg mice is interesting, since this may provide a potentially modifiable local cellular source of IGF-1 in the Aβ plaque-burdened brain in individuals with AD.

## Ethics Statement

Experiments were conducted according to permission from the Danish Ethical Animal Care Committee (Permission Nos. 2011/562-67 and 2011/561-1950).

## Author Contributions

CM conceptualized processing of mice, hippocampal plaque-load estimation, BrdU staining/quantification, qPCR, ISH, and writing of the manuscript. CT and BV performed cell culture work, qPCR, IHC of tissue, and quantitation of ISH. LI, JV, KK, MG, SZ, AK, and LD-O carried out the processing of mice and *ISH*. MJ, AB, and BF guided and supervised the study. BF initiated the project. All authors read, edited, and approved the manuscript.

## Conflict of Interest Statement

The authors declare that the research was conducted in the absence of any commercial or financial relationships that could be construed as a potential conflict of interest.

## Supplementary Material

FIGURE S1 *ISH* showing representative TNF mRNA^+^ cells in the hippocampus of 12- and 24-month-old WT and APP_swe_/PS1_ΔE9_ Tg mice. Scale bar: 10 μm.

FIGURE S2 Distribution of IGF-1 mRNA^+^ cells in postnatal day five (P5) old mouse pups. *ISH* of horizontal sections of P5 C57BL/6 mouse pups with AP-conjugated probe for IGF-1 mRNA. **(A)** High power photomicrograph of IGF-1 mRNA^+^ cells with the morphology of amoeboid microglia in the corpus callosum. Overview of the hippocampal formation (HF), and high power of IGF-1 mRNA^+^ cells in the angular bundle and IGF-1 mRNA^+^ sgz cells at the border between the granule cell layer and the dentate hilus (DG). **(B)** ISH showing IGF-1 mRNA^+^ cells in the hippocampus (higher magnification of section shown in **(A)**, and blank RNAse control (RNAse) and competition control (Comp) in parallel sections from the same mouse. ab, angular bundle; BS, brain stem; CA1, CA3, regio superior and inferior hippocampus, respectively; cc, corpus callosum; DG, dentate gyrus; ec, external capsule; f, fimbria-fornix; h, hilus; g, granule cell layer; ml, molecular layer; and Th, thalamus. Scale bars: 150 μm **(A,C,D)**, 250 μm **(B)**, 250 μm **(E–G)**.

FIGURE S3 IGF-1 expression in cerebellar and hippocampal neurons. IHC for IGF-1 on sections from 24-month-old APP_swe_/PS1_ΔE9_ Tg and 26-month-old WT mice. IHC was performed by use of a two-step protocol using AP-conjugated anti-rabbit antibody for the detection of the primary rabbit-anti-IGF-1 antibody. **(A)** IGF-1 is expressed in high levels in cerebellar Purkinje cells. **(B)** In the hippocampal formation, IGF-1 immunoreactivity is most abundant in entorhinal layer II neurons and in fibre-like structures in the CA3 pyramidal cell layer. Substitution control performed with inert rabbit IgG abolished all staining. CA3, regio superior hippocampus; CB, cerebellum; and HF, hippocampal formation. Scale bars: 50 μm (**A**, bottom), 100 μm (**A**, top and **B**, bottom), and 400 μm (**A**, top).

FIGURE S4 Double-immunofluorescence staining for IGF-1 and microglial CD11b in dentate gyrus. **(A)** Single-layers showing of the immunofluorescence signal for CD11b (red) and IGF-1 (green) in the dentate gyrus (DG) of 24-month-old APP_swe_/PS1_ΔE9_, corresponding to Figure 5B. Nuclei are stained with DAPI (blue). Fresh frozen sections were used for the double-immunofluorescence stainings. Pictures were obtained using a 40 × objective creating a z-stack through the section. **(B)** Orthogonal views of the z-stack showing overlap of IGF-1 and CD11b immunofluorescense signal (yellow). Scale bars: 20 μm.

FIGURE S5 Double-immunofluorescence staining for IGF-1 and microglial CD11b in the entorhinal cortex. **(A)** Single-layers showing of the immunofluorescence signal for CD11b (red) and IGF-1 (green) in the entorhinal cortex (EC) of 24-month-old APP_swe_/PS1_ΔE9_, corresponding to Figure 5B. Nuclei are stained with DAPI (blue). Fresh frozen sections were used for the double-immunofluorescence stainings. Pictures were obtained using a 40 × objective creating a z-stack through the section. **(B)** Orthogonal views of the z-stack showing overlap of IGF-1 and CD11b immunofluorescense signal (yellow). Scale bars: 20 μm.

FIGURE S6 Double-immunofluorescence staining for IGF-1 and microglial CD11b in the neocortex. **(A)** Single-layers showing of the immunofluorescence signal for CD11b (red) and IGF-1 (green) in neocortex (NCX) of 24-month-old APP_swe_/PS1_ΔE9_, corresponding to Figure 5B. Nuclei are stained with DAPI (blue). Fresh frozen sections were used for the double-immunofluorescence stainings. Pictures were obtained using a 40 × objective creating a z-stack through the section. **(B)** Orthogonal views of the z-stack showing overlap of IGF-1 and CD11b immunofluorescense signal (yellow). Scale bars: 20 μm.

FIGURE S7 Double-immunofluorescence stainings for IGF-1 and CD11b in sections from 24-month-old APP_swe_/PS1_ΔE9_ mice. IGF-1 (green), CD11b (red), and nuclei are stained with DAPI (blue). **(A)** Amyloid-plaque-associated aggregates of CD11b^+^ microglia (arrows) are abundant in the entorhinal cortex. Note, the high levels of IGF-1 expression in entorhinal layer II neurons. **(B)** Substitution control performed on section to the one used for the photomicrographs shown in **(A,C,D)**. Substitution of both primary antibodies with inert rabbit IgG and rat IgG2b, respectively, did not abolish all signal, but left behind a faint vascular staining from the secondary AlexaFluor568 goat-anti rat IgG. **(C)** Cerebellar cortex with IGF-1 expression in the Purkinje cells (arrows). **(D)** IGF-1 immunoreactive fibre-like structures between the CA3 pyramidal cells. CB, cerebellum; EC, entorhinal cortex; g, granule cell layer; ml, molecular layer; o, stratum oriens; p, pyramical cell layer; r, stratum radiatum; and wm, white matter. Scale bars: 100 μm **(A)**, 100 μm **(B)**, and 20 μm **(C,D)**.

FIGURE S8 BDNF mRNA levels do not change with age in APP_swe_/PS1_ΔE9_ Tg and WT mice. Quantitative PCR analysis shows that levels of BDNF mRNA are not significantly different with age in hippocampus of either Tg mice (gray bars) or WT mice (white bars). Specific age groups are indicated on the graph. Bars represent medians with 25 and 75% quartiles (*n* = 6–10 per group).

TABLE S1 Summary of stereology data on % Aβ plaque load and hippocampal volume in APP_swe_/PS1_ΔE9_ Tg mice.

TABLE S2 IGF-1 mRNA expression by CD11b mRNA^+^ microglia in the molecular layer of aged WT and APP_swe_/PS1_ΔE9_ Tg mice.
