# Supplementary figures and images for: Microglia Express Insulin-Like Growth Factor-1 in the Hippocampus of Aged APPswe/PS1ΔE9 Transgenic Mice
Source: Front Cell Neurosci. 2019 Jul 30;13:308. doi: 10.3389/fncel.2019.00308 (PMC6682662; doi:10.3389/fncel.2019.00308)

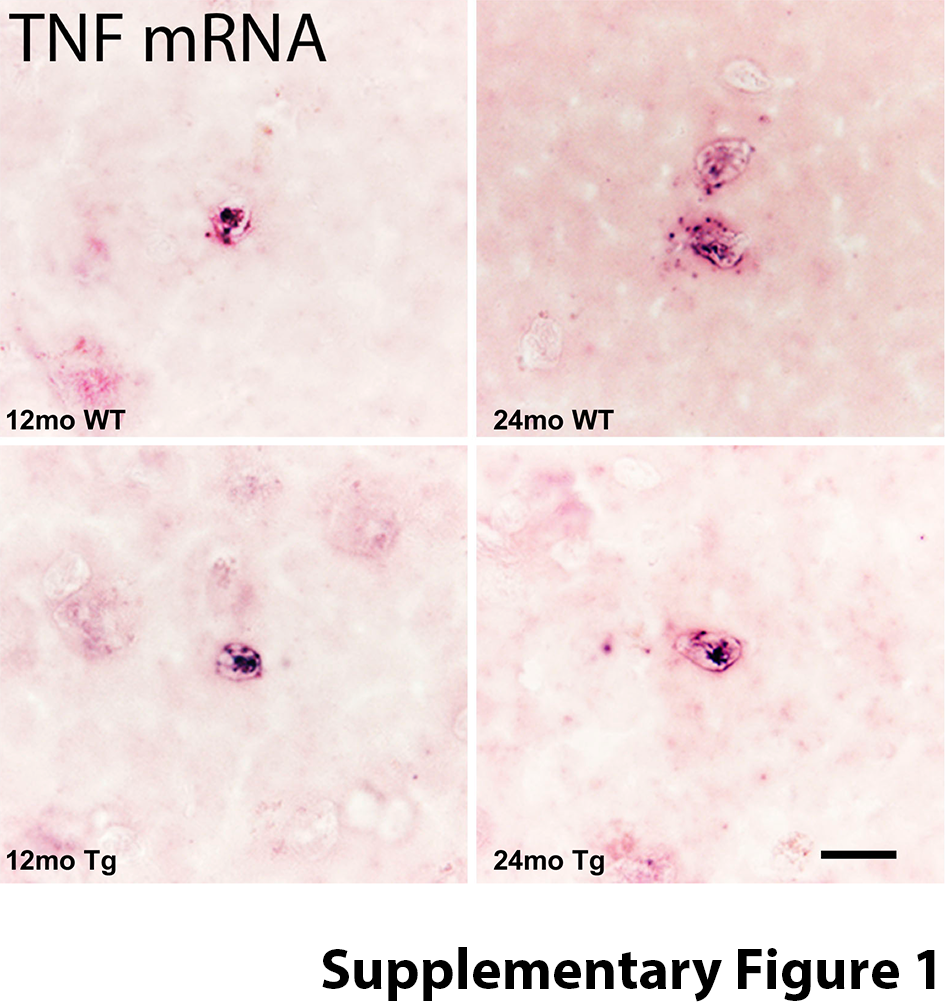

Supplement: FIGURE S1 — ISH showing representative TNF mRNA+ cells in the hippocampus of 12- and 24-month-old WT and APPswe/PS1ΔE9 Tg mice. Scale bar: 10 μm. [file Image_1.TIF]

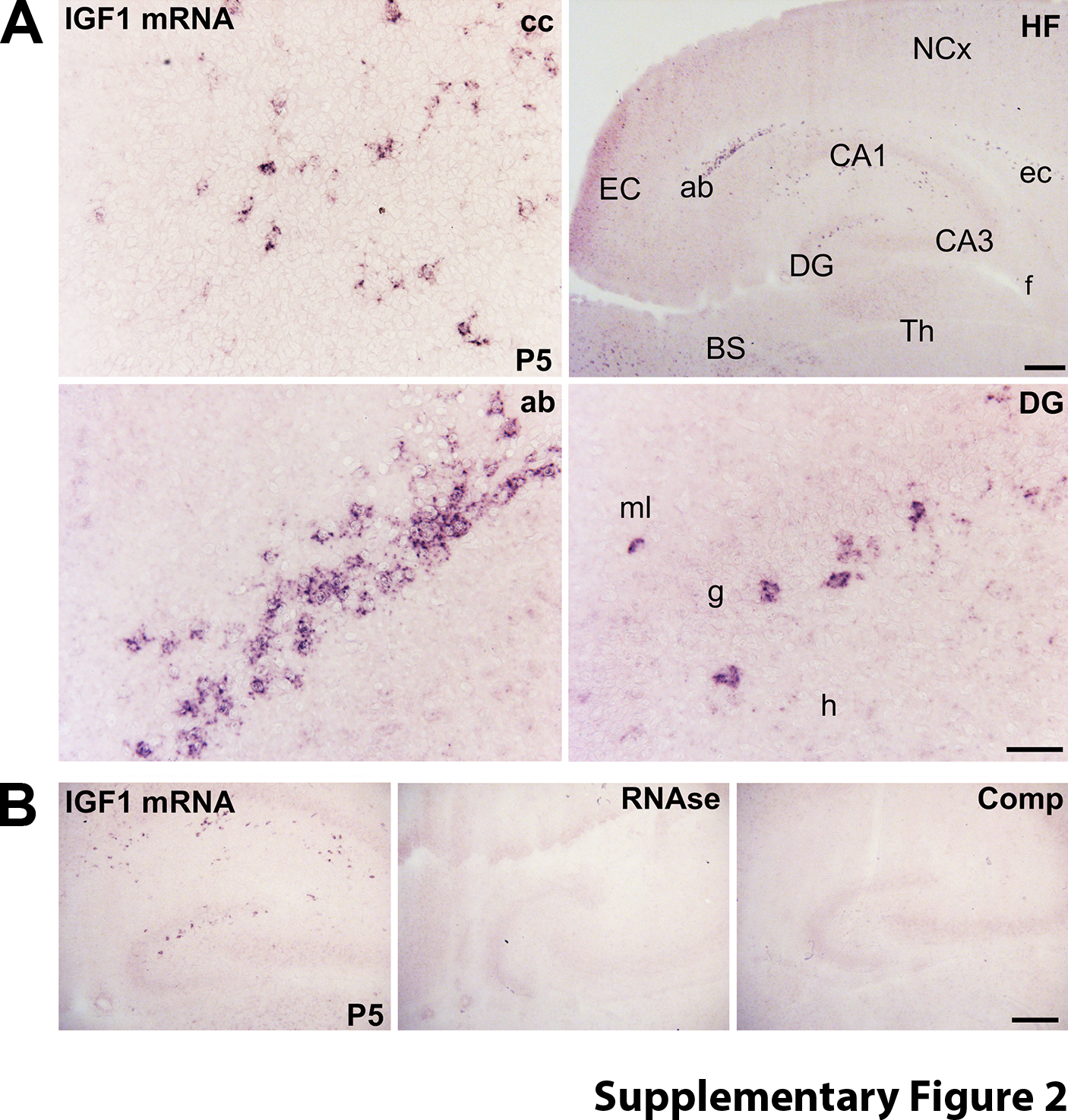

Supplement: FIGURE S2 — Distribution of IGF-1 mRNA+ cells in postnatal day five (P5) old mouse pups. ISH of horizontal sections of P5 C57BL/6 mouse pups with AP-conjugated probe for IGF-1 mRNA. (A) High power photomicrograph of IGF-1 mRNA+ cells with the morphology of amoeboid microglia in the corpus callosum. Overview of the hippocampal formation (HF), and high power of IGF-1 mRNA+ cells in the angular bundle and IGF-1 mRNA+ sgz cells at the border between the granule cell layer and the dentate hilus (DG). (B) ISH showing IGF-1 mRNA+ cells in the hippocampus (higher magnification of section shown in (A), and blank RNAse control (RNAse) and competition control (Comp) in parallel sections from the same mouse. ab, angular bundle; BS, brain stem; CA1, CA3, regio superior and inferior hippocampus, respectively; cc, corpus callosum; DG, dentate gyrus; ec, external capsule; f, fimbria-fornix; h, hilus; g, granule cell layer; ml, molecular layer; and Th, thalamus. Scale bars: 150 μm (A,C,D), 250 μm (B), 250 μm (E–G). [file Image_2.TIF]

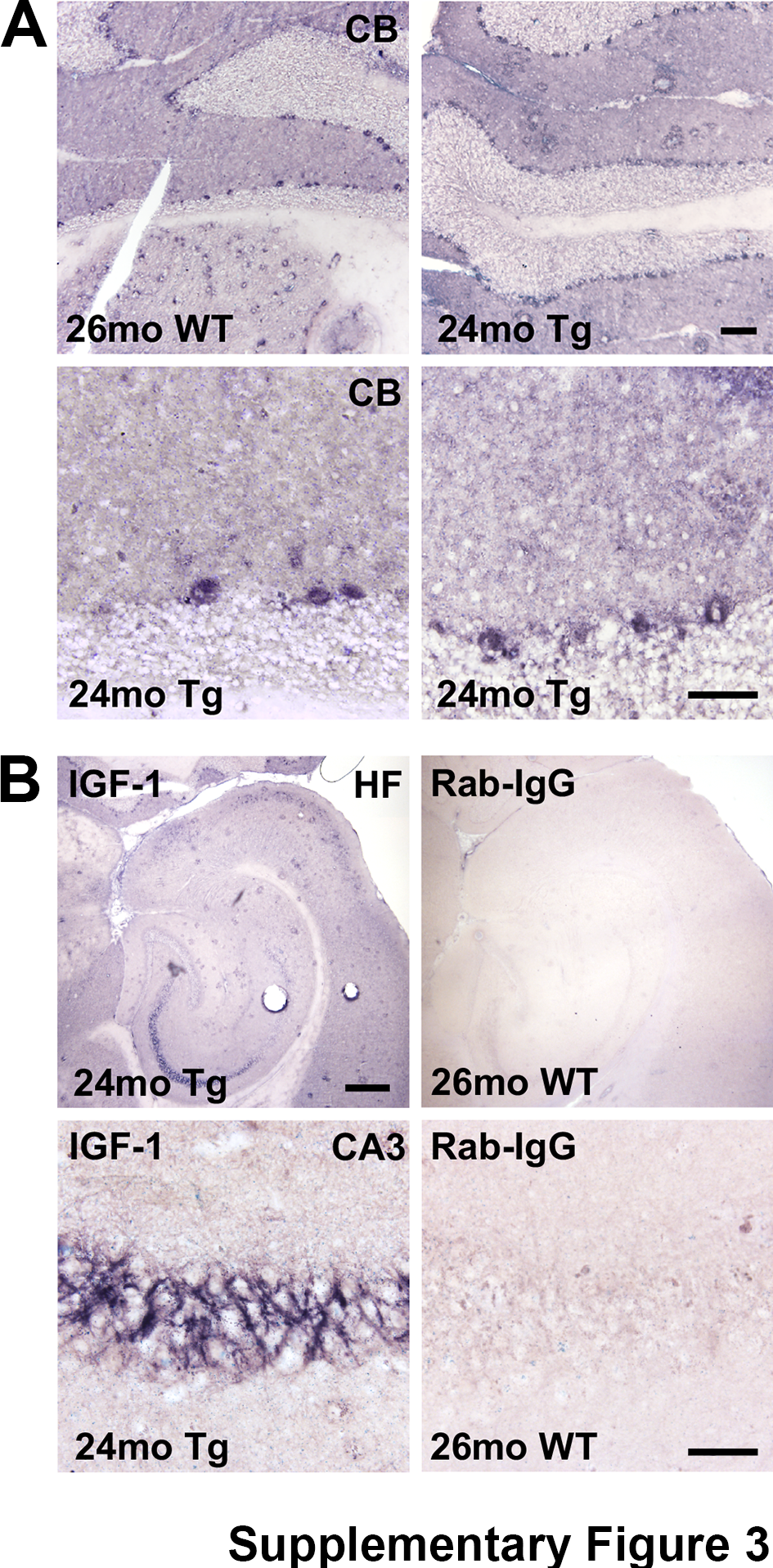

Supplement: FIGURE S3 — IGF-1 expression in cerebellar and hippocampal neurons. IHC for IGF-1 on sections from 24-month-old APPswe/PS1ΔE9 Tg and 26-month-old WT mice. IHC was performed by use of a two-step protocol using AP-conjugated anti-rabbit antibody for the detection of the primary rabbit-anti-IGF-1 antibody. (A) IGF-1 is expressed in high levels in cerebellar Purkinje cells. (B) In the hippocampal formation, IGF-1 immunoreactivity is most abundant in entorhinal layer II neurons and in fibre-like structures in the CA3 pyramidal cell layer. Substitution control performed with inert rabbit IgG abolished all staining. CA3, regio superior hippocampus; CB, cerebellum; and HF, hippocampal formation. Scale bars: 50 μm (A, bottom), 100 μm (A, top and B, bottom), and 400 μm (A, top). [file Image_3.TIF]

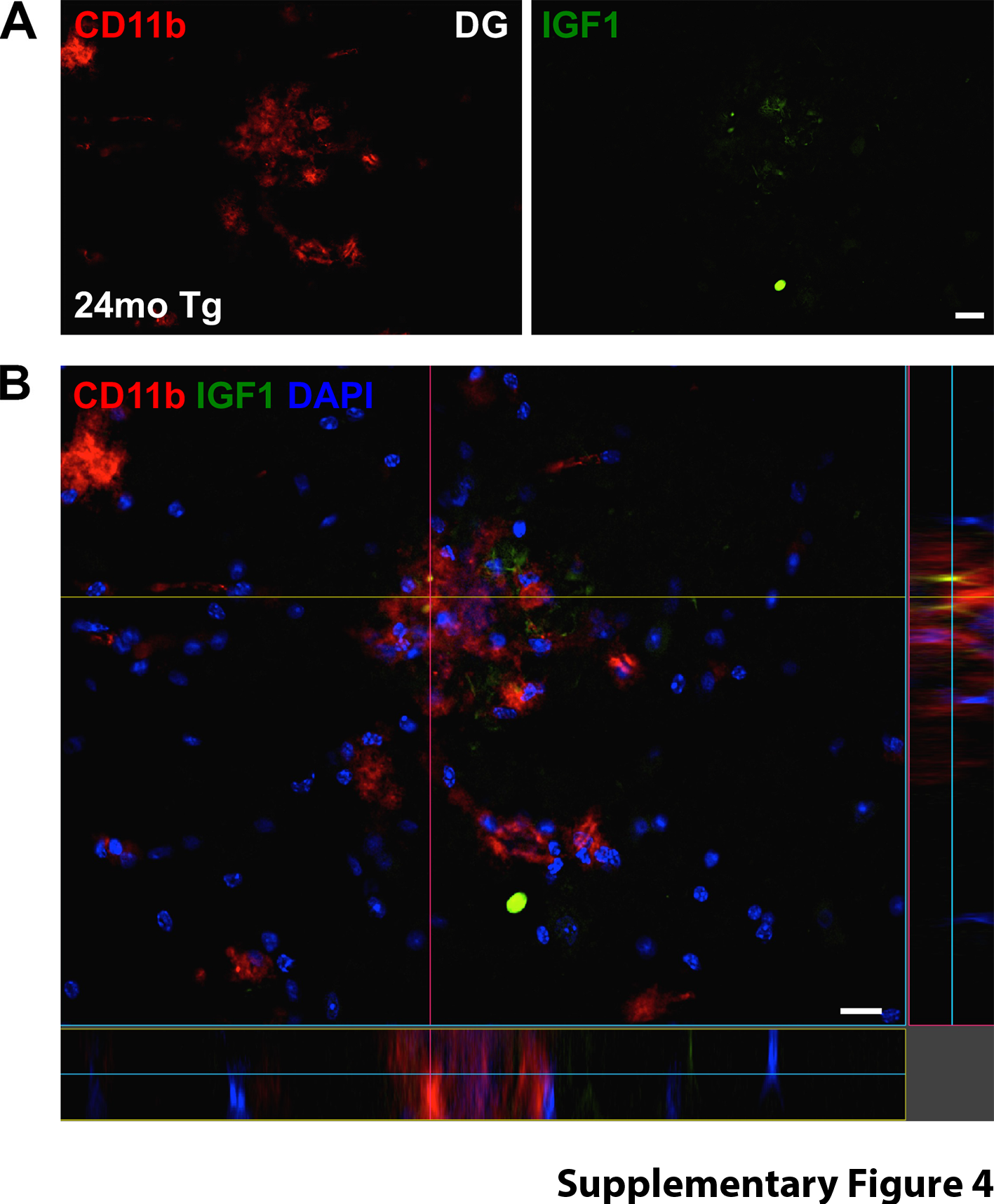

Supplement: FIGURE S4 — Double-immunofluorescence staining for IGF-1 and microglial CD11b in dentate gyrus. (A) Single-layers showing of the immunofluorescence signal for CD11b (red) and IGF-1 (green) in the dentate gyrus (DG) of 24-month-old APPswe/PS1ΔE9, corresponding to Figure 5B. Nuclei are stained with DAPI (blue). Fresh frozen sections were used for the double-immunofluorescence stainings. Pictures were obtained using a 40 × objective creating a z-stack through the section. (B) Orthogonal views of the z-stack showing overlap of IGF-1 and CD11b immunofluorescense signal (yellow). Scale bars: 20 μm. [file Image_4.TIF]

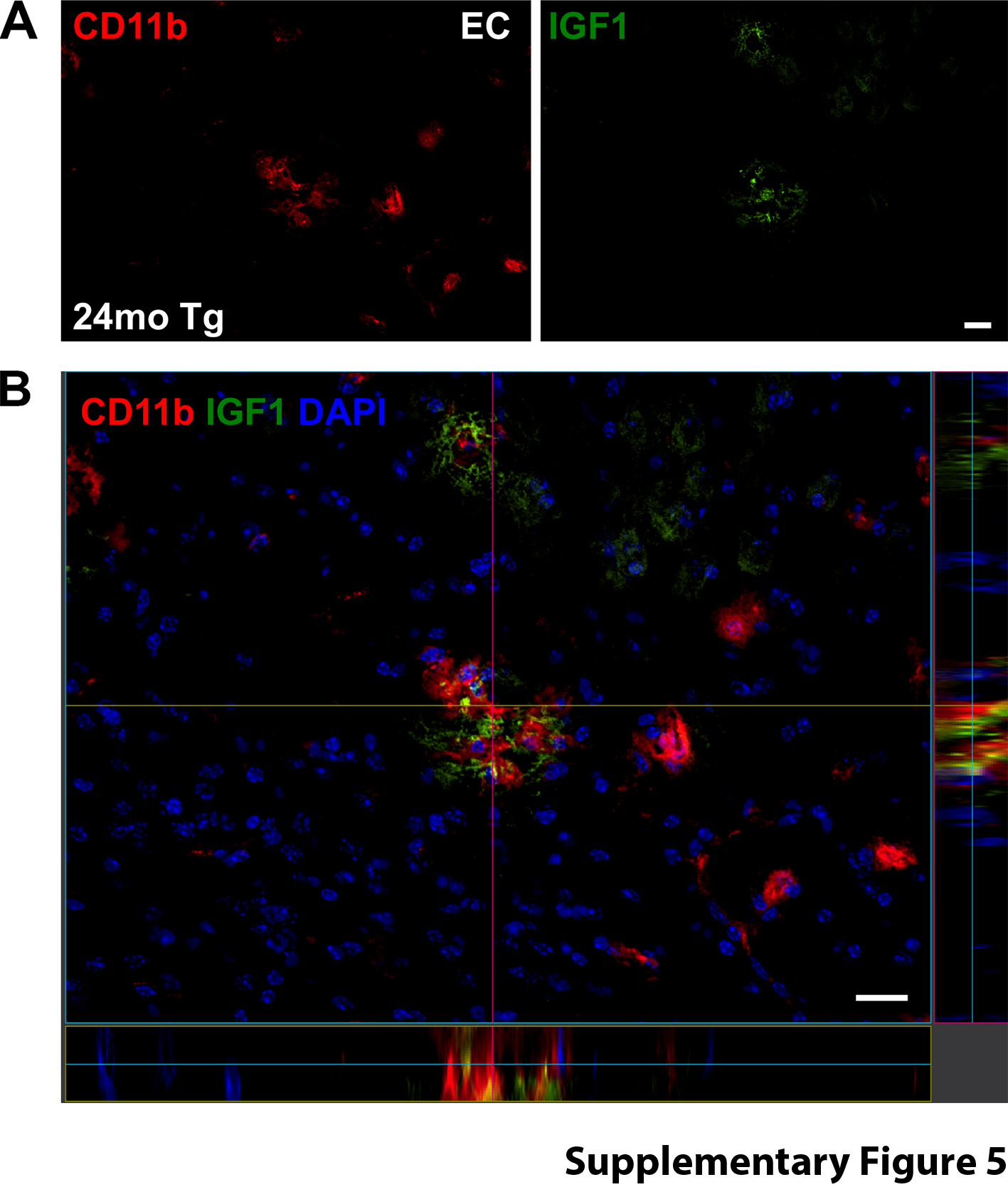

Supplement: FIGURE S5 — Double-immunofluorescence staining for IGF-1 and microglial CD11b in the entorhinal cortex. (A) Single-layers showing of the immunofluorescence signal for CD11b (red) and IGF-1 (green) in the entorhinal cortex (EC) of 24-month-old APPswe/PS1ΔE9, corresponding to Figure 5B. Nuclei are stained with DAPI (blue). Fresh frozen sections were used for the double-immunofluorescence stainings. Pictures were obtained using a 40 × objective creating a z-stack through the section. (B) Orthogonal views of the z-stack showing overlap of IGF-1 and CD11b immunofluorescense signal (yellow). Scale bars: 20 μm. [file Image_5.TIF]

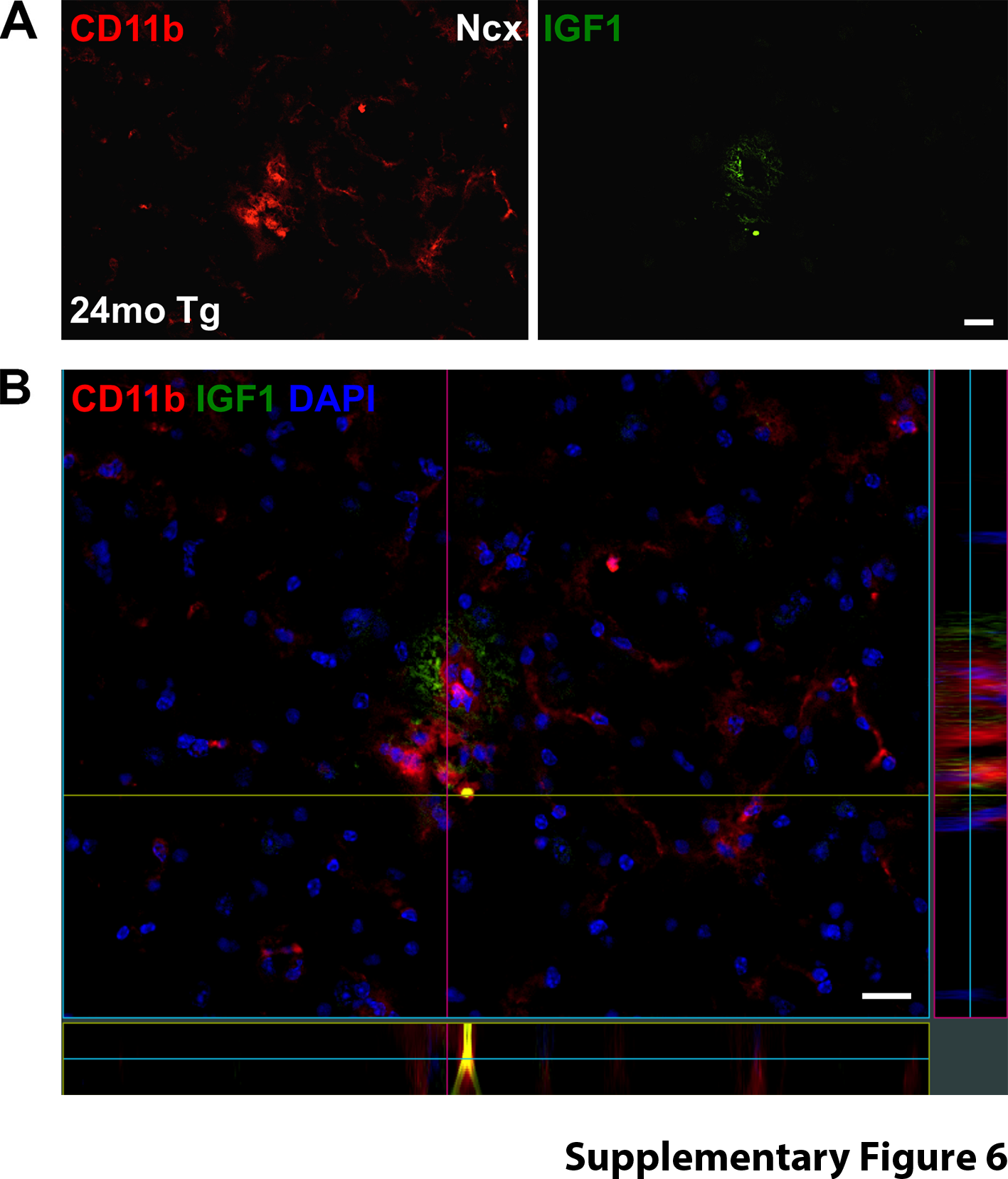

Supplement: FIGURE S6 — Double-immunofluorescence staining for IGF-1 and microglial CD11b in the neocortex. (A) Single-layers showing of the immunofluorescence signal for CD11b (red) and IGF-1 (green) in neocortex (NCX) of 24-month-old APPswe/PS1ΔE9, corresponding to Figure 5B. Nuclei are stained with DAPI (blue). Fresh frozen sections were used for the double-immunofluorescence stainings. Pictures were obtained using a 40 × objective creating a z-stack through the section. (B) Orthogonal views of the z-stack showing overlap of IGF-1 and CD11b immunofluorescense signal (yellow). Scale bars: 20 μm. [file Image_6.TIF]

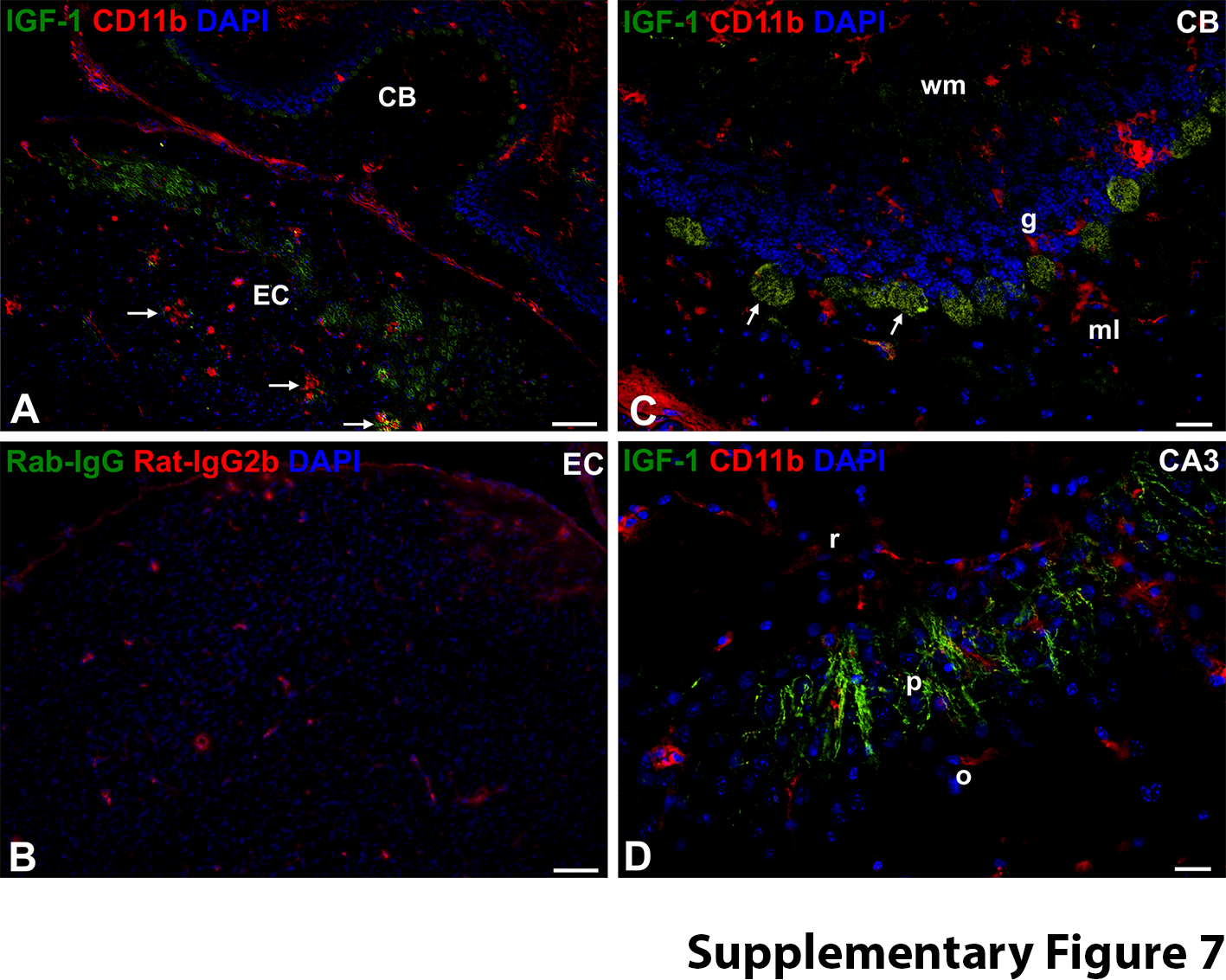

Supplement: FIGURE S7 — Double-immunofluorescence stainings for IGF-1 and CD11b in sections from 24-month-old APPswe/PS1ΔE9 mice. IGF-1 (green), CD11b (red), and nuclei are stained with DAPI (blue). (A) Amyloid-plaque-associated aggregates of CD11b+ microglia (arrows) are abundant in the entorhinal cortex. Note, the high levels of IGF-1 expression in entorhinal layer II neurons. (B) Substitution control performed on section to the one used for the photomicrographs shown in (A,C,D). Substitution of both primary antibodies with inert rabbit IgG and rat IgG2b, respectively, did not abolish all signal, but left behind a faint vascular staining from the secondary AlexaFluor568 goat-anti rat IgG. (C) Cerebellar cortex with IGF-1 expression in the Purkinje cells (arrows). (D) IGF-1 immunoreactive fibre-like structures between the CA3 pyramidal cells. CB, cerebellum; EC, entorhinal cortex; g, granule cell layer; ml, molecular layer; o, stratum oriens; p, pyramical cell layer; r, stratum radiatum; and wm, white matter. Scale bars: 100 μm (A), 100 μm (B), and 20 μm (C,D). [file Image_7.TIF]

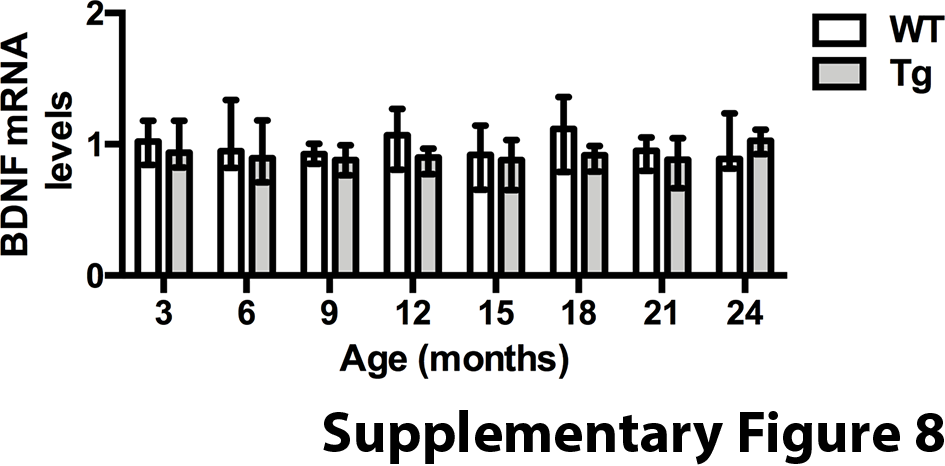

Supplement: FIGURE S8 — BDNF mRNA levels do not change with age in APPswe/PS1ΔE9 Tg and WT mice. Quantitative PCR analysis shows that levels of BDNF mRNA are not significantly different with age in hippocampus of either Tg mice (gray bars) or WT mice (white bars). Specific age groups are indicated on the graph. Bars represent medians with 25 and 75% quartiles (n = 6–10 per group). [file Image_8.TIF]
