# Supplementary material for: Microglia Express Insulin-Like Growth Factor-1 in the Hippocampus of Aged APPswe/PS1ΔE9 Transgenic Mice
Source: Front Cell Neurosci. 2019 Jul 30;13:308. doi: 10.3389/fncel.2019.00308 (PMC6682662; doi:10.3389/fncel.2019.00308)
Supplement: TABLE S1 — Summary of stereology data on % Aβ plaque load and hippocampal volume in APPswe/PS1ΔE9 Tg mice. [file Data_Sheet_1.docx]

**Supplementary Table 1. Summary of stereology data on % Aβ plaque load and hippocampal volume in APP_swe_/PS1_ΔE9_ Tg mice**

| **Analysis** | | **Age** | | | | | | | |
| --- | --- | --- | --- | --- | --- | --- | --- | --- | --- |
|  |  |  |  |  |  |  |  |  |  |
|  |  | **3 mo** | **6 mo** | **9 mo** | **12 mo** | **15 mo** | **18 mo** | **21 mo** | **24 mo** |
|  |  | n = 6 | n = 6 | n = 6 | n = 6 | n = 4 | n = 6 | n = 6 | n = 6 |
| **% Aβ plaque load** | **Median %**  (25%-75% quartiles) | 0.02  (0.01- 0.03) | 0.26  (0.02-0.32) | 0.91  (0.76-1.51) | 2.55  (2.28-2.9) | 4.98*  (3.87-6.19) | 4.68**  (3.87-6.19) | 6.11***  (4.50-7.49) | 6.37***  (5.09-6.80) |
|  | **Mean %** | 0.02 | 0.26 | 1.07 | 2.64^####^ | 4.66^####^ | 4.83^####^ | 6.05^####^ | 5.94^####^ |
|  | **SD** | 0.01 | 0.07 | 0.39 | 0.54 | 1.34 | 1.26 | 1.72 | 1.29 |
|  | ^a^**CE** | 0.68^c^ | 0.20 | 0.12 | 0.09 | 0.11 | 0.09 | 0.09 | 0.11 |
|  | ^b^**CV** | 0.36^c^ | 0.28 | 0.36 | 0.21 | 0.29 | 0.26 | 0.28 | 0.22 |
| **Hippocampal volume**^d^ |  | **3 mo**  n=5 | **6 mo**  n=6 | **9 mo**  n=5 | **12 mo**  n=6 | **15 mo**  n=4 | **18 mo**  n=5 | **21 mo**  n=6 | **24 mo**  n=3 |
|  | **Median (mm^3^)**  (25%-75% quartiles) | 10.9  (8,9-11,4) | 10,7  (9.9-12,1) | 10.4 (8.5-12.4) | 13.5  (11.0-15.4) | 11.3 (9.7-13.8) | 11.6 (10.7-12.2) | 11.6 (8.1-13.3) | 11.9  (8.6-11.9) |
|  | **Mean (mm^3^)** | 10.3 | 10.9 | 10.4 | 13.0 | 11.6 | 11.5 | 11.0 | 10.7 |
|  | **SD** | 1.3 | 1.10 | 1.9 | 2.8 | 2.2 | 0.8 | 2.4 | 1.7 |
|  | **CV** | 0.12 | 0.10 | 0.19 | 0.22 | 0.19 | 0.07 | 0.22 | 0.15 |

^a^The average coefficient of error (CE) is calculated as the mean of the individual CE estimates within each group. For additional details on the calculation of CE, see ([West 2012](#_ENREF_83); [West et al. 1996](#_ENREF_85)).

^b^The coefficient of variation, CV, is calculated as CV = SD/Mean.

^c^One 3-month-old mouse was excluded in the plaque (CE/CV) calculations since no plaques were counted.

^d^The hippocampal volume is estimated using the areas obtained from the Aβ quantification. The volume for individual age groups is given as the average volume from estimates of the mean volume calculated from two different series of sections.

*p<0.05, **p<0.01, ***p<0.001, compared to 3mo APP/PS1 Tg mice, by Kruskal-Wallis test.

^####^p<0.0001, compared to 3mo APP/PS1 Tg mice, by One-Way ANOVA with Bonferroni’s Multiple Comparison post-hoc test.

**Supplementary Table 2.** **IGF-1 mRNA expression by CD11b mRNA^+^ microglia in the molecular layer of aged WT and *APP_swe_/PS1_ΔE9_***  **Tg mice**

| **Genotype / Cellular subtypes &**  **Cellular mRNA expression** | **WT**^1^  Mean (Range) | **APP_swe_/PS1_ΔE9_** **Tg**^2^  Mean (Range) | **P-value**^3^ |
| --- | --- | --- | --- |
| **Cellular subtypes** | | | |
| IGF-1 mRNA^+^ cells (cells/mm^2^) | 108 (93-121) | 1. (84-289) | 0.57 |
| CD11b mRNA^+^ cells (cells/mm^2^) | 336 (250-504) | 693 (493-872) | 0.07 |
| IGF-1 mRNA^+^ CD11b mRNA^+^ cells (cells/mm^2^) | 48 (45-57) | 110 (71-165) | 0.036 |
| **Cellular mRNA expression** | | | |
| IGF-1 mRNA puncta / CD11b mRNA^+^ cell  (No. of cells quantified) | 0.30 (0.3-0.4)  (25) | 0.35 (0.3-0.4)  (138) | 0.43 |
| CD11b mRNA puncta / cell  (No. of cells quantified) | 2.6 (1.7-3.6)  (177) | 3.1 (3.0-3.4)  (1031) | 0.58 |
| **Area quantified per mouse** | | | |
| Area in mm^2^ | 0.17 (0.16-0.17) | 0.28 (0.19-0.34) | N/A |

^1^ Two mice per group (3 hippocampi), ^2^ Three mice per group (5 hippocampi), ^3^Mann-Whitney test. N/A, not applicable.
